# Injectable and Sprayable Thermoresponsive Hydrogel with Fouling‐Resistance as an Effective Barrier to Prevent Postoperative Cardiac Adhesions

**DOI:** 10.1002/advs.202500731

**Published:** 2025-03-28

**Authors:** Kun Shi, Tao Li, Xulin Hu, Wen Chen, Yan Yu, Zhongwu Bei, Liping Yuan, Qi Tong, Jiafeng Liu, Qiang Fan, Yongjun Qian, Zhiyong Qian

**Affiliations:** ^1^ Department of Biotherapy Cancer Center and State Key Laboratory of Biotherapy West China Hospital Sichuan University Chengdu 610041 P. R. China; ^2^ Department of Pediatric Cardiac Surgery West China the Second Hospital Sichuan University Chengdu 610041 P. R. China; ^3^ Department of Cardiovascular Surgery National Clinical Research Center for Geriatrics West China Hospital Sichuan University Chengdu Sichuan 610041 P. R. China; ^4^ Clinical Medical College and Affiliated Hospital of Chengdu University Chengdu University Chengdu Sichuan 610081 P. R. China

**Keywords:** adhesiolysis, adhesion prevention, fouling‐resistance, postoperative cardiac adhesion, thermo‐responsive hydrogel

## Abstract

Postoperative adhesions are inevitable consequences of surgery, resulting in various complications. The risk increases, particularly in cardiac surgery, where reoperations or staged operations are in high demand. The presence of cardiac adhesions severely complicates resternotomy procedures, which may increase the risk of re‐traumatizing, bleeding, operation time extension, and even mortality. Here, a biodegradable thermo‐sensitive fouling‐resistant hydrogel based on poly(D,L‐lactide)/poly(ethylene glycol) (PLEL) micelles is developed, exhibiting high compatibility with both thoracotomies and minimally invasive interventions. By combining the advantages of liquid and film barriers, this hydrogel can completely cover irregular wounds after injecting or spraying and fully avoid direct contact with injuries between adjacent wound surfaces during the critical course of adhesion formation. In both the primary cardiac adhesion model and the severe repeated‐injury cardiac adhesion model, the PLEL hydrogel is more effective than commercially available products (including Interceed film and sodium hyaluronate hydrogel) in significantly reducing the incidence and severity of adhesions, without causing any cardiac dysfunction or organ damage. The powerful anti‐adhesion effect of PLEL hydrogel is closely related to its ability to inhibit the inflammatory response and balance the fibrinolysis system. This injectable and sprayable antifouling thermosensitive hydrogel represents a promising clinical solution for the prevention of postoperative and recurrent cardiac adhesion.

## Graphical abstract

Postoperative cardiac adhesions lead to numerous difficulties in reoperation procedures. Here, a thermo‐responsive in situ formed hydrogel is shown to can reliably cover the wounds after a convenient spraying or injection and significantly reduce adhesion formation in primary and recurrent cardiac injury adhesion models. The simple preparation, easy administration, fast gelation, suitable retention time, fouling‐resistant ability, and increased adhesion prevention efficacy make the PLEL hydrogel a promising candidate for post‐operative cardiac adhesion prevention.

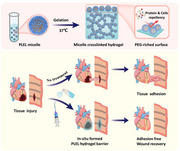

## 1 Introduction

The formation of adhesions following abdominopelvic and thoracic surgical interventions is one of the most common and challenging medical problems, as up to 93% of patients suffer this complication after the operation.^[^ ^1^, ^2^ ^]^ It may lead to severe or chronic pain and organ dysfunction, resulting in 15%–30% of patients requiring adhesiolysis to release the adhesions. Even after adhesiolysis, the incidence of more serious recurrent adhesions remains high due to new trauma caused by surgical lysis.^[^ ^3^, ^4^ ^]^ This problem is particularly relevant in cardiac surgery, in which secondary surgery or staged operations are in high demand. Children born with congenital heart defects commonly experience multiple redo cardiac surgeries over their lifetime and 10%–20% of the patients who undergo cardiac surgeries, such as coronary artery bypass grafting, and valve repair or replacement, need reoperation.^[^ ^5^, ^6^ ^]^ The presence of adhesions between the epicardium and other tissues in the chest cavity substantially prolongs reoperative time and raises the potential clinical risks of visibility obstruction, massive bleeding, and injury to the heart, vessels, and lungs during sternal re‐entry and cardiac dissection, leading to increased perioperative mortality.^[^ ^7^, ^8^ ^]^ Overall, post‐operative adhesion markedly increases the suffering of patients and has caused an enormous economic burden on healthcare, thus presenting an urgent clinical need for effective and safe anti‐adhesion measures to prevent postoperative and recurrent cardiac adhesion.

Current anti‐adhesion strategies include pharmacological treatments, barrier‐based devices, and even drug‐loaded barriers.^[^ ^9^, ^10^, ^11^ ^]^ Among them, physical barriers have been widely accepted to prevent adhesion by keeping the wound separate from the surrounding tissue during the early healing phase. The two most common commercially available anti‐adhesion products are solid films, Seprafilm (sodium hyaluronate‐carboxymethyl cellulose; Genzyme) and Interceed (oxidized regenerated cellulose; Johnson & Johnson), which are indicated for use only in the abdomen and have a low application rate in candidate surgeries.^[^ ^4^, ^12^ ^]^ Even if one film product named REPEL‐CV (polyethylene glycol/ polylactic acid sheet) was approved for preventing cardiac adhesion in the United States, it was found to reduce the severity but not the incidence of adhesion and failed to reduce adhesion dissection time.^[^ ^13^, ^14^ ^]^ One of the primary drawbacks of these film‐based products is that it is difficult to handle and fix these brittle films on the surface of a wound and completely cover entire damaged tissues with irregular shapes or heavily folded surfaces, such as the small intestine and great vessels of the heart. In addition, the inability to be used in minimally invasive laparoscopic, thoracoscopic, or catheter‐based procedures is another important reason for the decreased clinical adoption of film products.^[^ ^15^ ^]^ Polymer solutions such as an icodextrin solution (Adept) and a hyaluronic acid solution (Sepracoat) were found to address the shortages of film barriers to some extent, but the short residence time on the treated surface greatly compromises their effectiveness.^[^ ^16^, ^17^ ^]^ Therefore, preventing postsurgical cardiac adhesions still remains a major challenge in clinical treatment, and better anti‐adhesion barriers are needed.

In‐situ formed hydrogels combining the advantages of liquid and film barriers have the potential for use in both open and minimally invasive surgeries and may be particularly attractive as a substitute for commercially available anti‐adhesion barriers.^[^ ^18^, ^19^ ^]^ After being injected or sprayed on the surface of the wounds, the gel precursor solutions can gel in situ via chemical polymerization, such as Michael addition, Schiff base, and photo‐initiated radical crosslinking, or through a solution‐to‐hydrogel (sol‐gel) phase transition under mild conditions, such as temperature, pH value, and ionic concentration.^[^ ^20^, ^21^, ^22^, ^23^ ^]^ CoSeal surgical sealant ((polyethylene glycol (PEG) hydrogel) has been approved to prevent the formation of postoperative cardiac adhesions in some countries. Despite its effectiveness in reducing the severity of cardiac adhesions, the application of CoSeal hydrogel is limited by excessive swelling, which can lead to cardiac tamponade.^[^ ^24^ ^]^ In contrast, physical hydrogels, such as thermo‐sensitive ones, exhibit spontaneous sol‐gel behavior in response to the physiological conditions without the need for any extra additives or chemical reactions, resulting in greater safety and superiority as anti‐adhesion barriers.^[^ ^25^, ^26^ ^]^ Although hydrogel barriers have received considerable attention, most of them are under development, and it is challenging to design an ideal barrier for preventing cardiac adhesion because the following requirements should be met: 1) be injectable or sprayable to be compatible with minimally invasive procedures; 2) rapidly gel on the surface of tissue; 3) have proper degradation rate and enough retention time during the healing phase; 4) be minimal swelling without the risk of mechanical compression of the heart; 5) have befitting viscoelasticity to enable organs and tissues to move freely, especially the beating heart; and 6) be biocompatible.^[^ ^4^, ^14^, ^18^ ^]^

**Figure 1:**
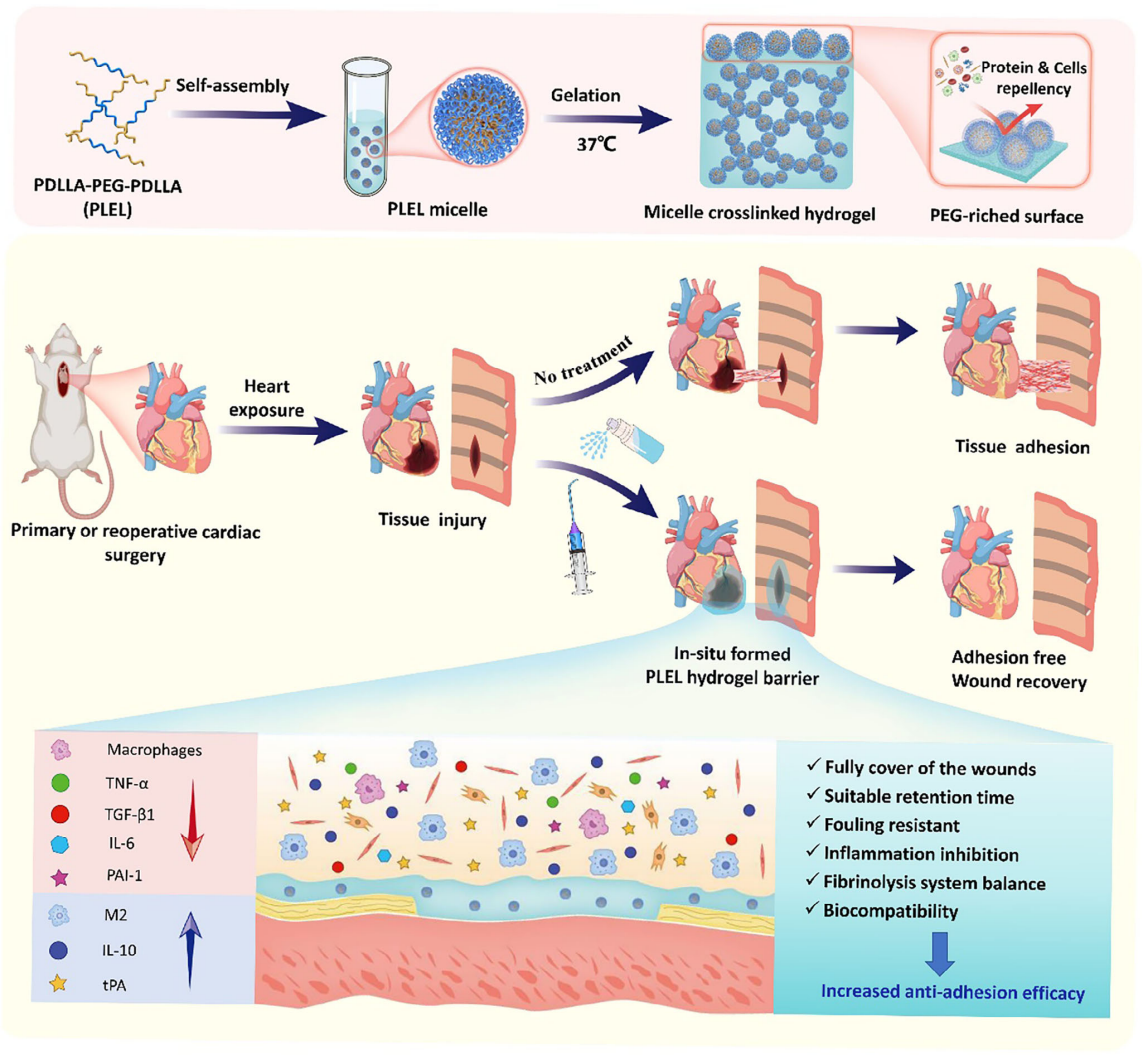
Schematic representation of an in situ formed thermo‐sensitive copolymer hydrogel with antifouling property, which can be injected or sprayed onto the surface of trauma to prevent the cardiac adhesion after primary or reoperative surgery by avoiding the contact of adjacent wounds, inhibiting the inflammatory response and balancing the fibrinolysis system.

We previously developed an injectable thermosensitive physical hydrogel made of a biodegradable amphiphilic triblock copolymer, poly(D,L‐lactide)‐poly(ethylene glycol)‐poly(D,L‐lactide) (PDLLA‐PEG‐PDLLA, PLEL), which displays rapid gelation in‐situ once exposed to the body temperature.^[^ ^27^ ^]^ Because of the amphiphilicity of PLEL triblock copolymers, they could self‐assemble into core‐shell‐like micelles in water with hydrophobic PDLLA cores and hydrophilic PEG shells. The micellar aggregation upon heating is thought to be involved in the underlying mechanism of the thermally induced sol‐gel transition. It has been well recognized that the deposition of proteins and adhesion of cells on the traumatized surface play an important role in the development of tissue adhesion, and materials coated with PEG polymers possess biological anti‐fouling properties to resist the adhesion of proteins and cells.^[^ ^28^, ^29^ ^]^ Therefore, we hypothesized that full prevention of postoperative cardiac adhesion could be accomplished by applying the PEG‐riched PLEL hydrogel as a barrier, which can not only avoid the direct contact of injured tissues but also inhibit the adhesion of proteins and cells in the critical formation course of adhesion (as shown in **Figure** **1**). Unlike most covalently crosslinked chemical hydrogels, PLEL hydrogel showed a reduced swelling ratio, contributing to minimizing the risk of cardiac tamponade and mechanical compression to the heart. Furthermore, the good fluidity of the PLEL solution at room temperature allows a facile application via multiple delivery methods, such as simple spraying or spreading to uniformly cover large and irregular areas in thoracotomies, injection through minimally invasive thoracoscopic methods, or delivery via catheter procedures.^[^ ^4^, ^15^ ^]^ In addition, the simplicity of its preparation through a one‐step ring‐opening copolymerization without the use of any possibly toxic coupling agent or organic solvent enables easy scale‐up manufacturing of PLEL hydrogel. On the basis of the rapid in‐situ gelation, anti‐fouling capacity, wide compatibility with different surgical procedures, and excellent biocompatibility of the PLEL hydrogel, we investigated its effectiveness for post‐operative cardiac adhesion prevention in two different challenging rat models (cardiac injury adhesion model and repeated‐injury adhesion model) compared with those of commercially available products, and the potential anti‐adhesion mechanism was studied in detail as well.

## 2 Results and Discussion

### 2.1 Preparation and Characterization of Thermo‐Sensitive PLEL Hydrogel

A biodegradable PDLLA‐PEG‐PDLLA (PLEL) copolymer was synthesized via the ring‐opening copolymerization of D,L‐LA initiated by PEG and catalyzed by stannous octoate (Sn(Oct)_2_). According to the results of the nuclear magnetic resonance spectroscopy (^1^H‐NMR) and gel permeation chromatography (GPC) measurements (Figure S1, Supporting Information), the number‐average molecular weight (Mn) of the obtained PLEL was 4764 (PEG/PDLLA ratio: 1500: 3264) and showed a unimodal distribution with a polydispersity index (PDI) of 1.63, indicating the successful synthesis of the PLEL copolymer.^[^ ^27^ ^]^ Notably, the preparation process of PLEL is straightforward without the use of any toxic organic solvent, which enables easy scaling‐up and good biocompatibility.

**Figure 2:**
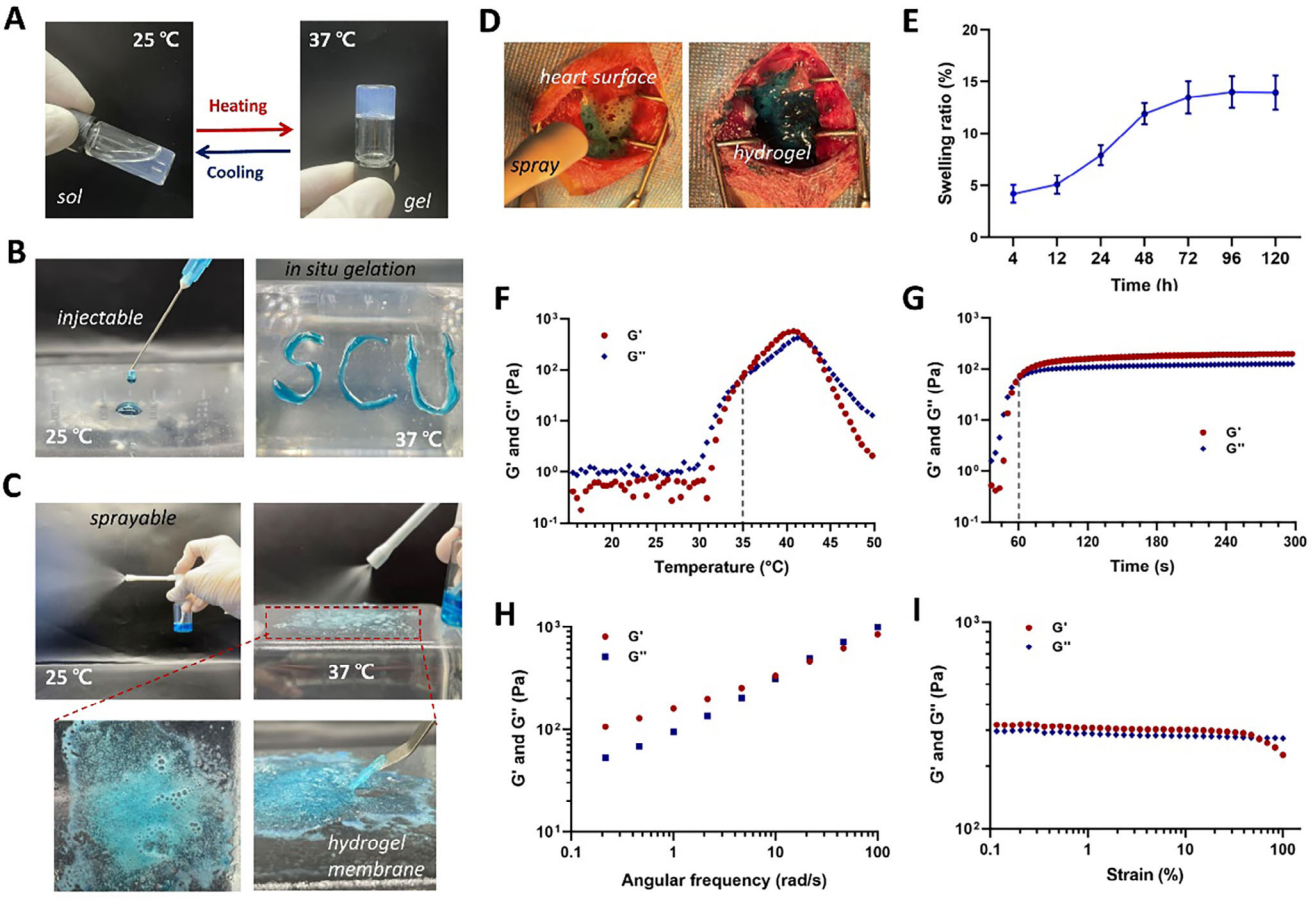
Characterization of injectable and sprayable thermo‐sensitive PLEL hydrogel. A) Photographs illustrating the sol‐gel phase transition of the PLEL solution between room temperature and body temperature. B) Injectability at room temperature and in situ rapid gelation at 37 °C of the PLEL solution. C) The PLEL solution can form a stable hydrogel membrane on the surface of a bottle filled with 37 °C water after spraying at room temperature. D) In situ gelation of blue dye‐loaded PLEL solution after spraying on the injured surface of a rat heart. E) Swelling ratio of the PLEL hydrogel when incubating in PBS at 37 °C. F) Changes in the storage (G') and loss modulus (G") of the PLEL hydrogel as a function of temperature. G) Gelation time study of the PLEL hydrogel via rheological tests. (H) Frequency‐dependent oscillatory sweep (under 1% strain, 37 °C). (I) Strain‐dependent oscillatory sweep (at a frequency of 10 rad s^−1^, 37 °C).

Owing to its amphiphilicity, PLEL triblock copolymers can self‐assemble into core‐shell‐like micelles in water. Transmission electronmicroscope (TEM) observation and dynamic light scattering (DLS) detection were conducted to characterize the morphology and size of PLEL micelles. As shown in Figure S2 (Supporting Information), the micelles were spherical nanoparticles with an average particle size of 43 nm. At room temperature, the PLEL micelles can flow freely, and their size augments with the increase in temperature. When the temperature rises to around body temperature, a micellar network can spontaneously form due to the sharp aggregation and packing between micelles, resulting in a physical hydrogel, as schematically presented in Figure 1 and shown in **Figure** **2**A.^[^ ^27^, ^30^ ^]^ In addition, the PLEL solution could be smoothly injected through a 22G syringe at room temperature and gelated rapidly on site when it touched the surface of a bottle filled with 37 °C water (Figure 2B). Figure 2C presented the spraying process of the PLEL solution via a medical throat spraying bottle, and the good fluidity at room temperature boded well for clinical administration. After being sprayed onto the 37 °C surface, a uniform hydrogel membrane was formed immediately with favorable self‐supporting ability. A similar in situ sol‐gel phase transition was observed when the PLEL solution was sprayed on the injured surface of the rat heart, suggesting the possibility of its use in vivo for preventing cardiac adhesion (Figure 2D). As shown in Figure 2E, the PLEL hydrogel presented good stability when incubated in PBS at 37 °C for several days, with a slight swelling ratio of less than 15%, suggesting that there is no risk of cardiac tamponade or mechanical compression of the heart in vivo.

The viscoelastic properties of the PLEL hydrogel were studied by rheological tests. The temperature sweep results revealed that both the storage modulus (G′) and loss modulus (G″) of the PLEL hydrogel increased sharply when the temperature exceeded 33 °C, and G' became dominant over G″ at ≈36 °C, indicating the formation of an elastic‐like gel, which was coincident with the macroscopic observations (Figure 2F). During the time sweep measurement, the sol‐gel phase transition of the PLEL hydrogel occurred within 65 s at 37 °C, followed by the maintaining of good mechanical stability, as shown in Figure 2G. Angular frequency‐dependent oscillatory sweeps in the linear viscoelastic regime (under 1% strain) showed that G' and G″ increased with increasing frequency over the entire test frequency range (0.1–100 rad/s). Moreover, the PLEL hydrogel maintained an elastic‐like behavior at a low frequency (< 20 rad/s), with G' higher than G″, but more viscous behavior at higher frequencies was observed (G′ < G″), where the micellar crosslinked network was gradually damaged (Figure 2H).^[^ ^28^ ^]^ In addition, strain‐dependent oscillatory rheological measurements were performed, and the results indicated that the PLEL hydrogel maintained solid‐like behavior in the low strain range (0.1%–50%), with G' dominating over G″, until fluid‐like behavior (G′ > G″) was exhibited when the strain further increased. It's reported that the highest strain experienced by tissues in the body is less than 10%, suggesting solid‐like behavior in the in vivo environment of our PLEL hydrogel.^[^ ^12^, ^31^ ^]^ Taken together, the thermosensitive PLEL hydrogel we developed has good injectability, feasible sprayability, a suitable phase inversion temperature and gelation time, a mild swelling ratio, and little effect on stability after gelation in vivo, implying great application potential as an adhesion prevention barrier.

### 2.2 In Vitro Cytocompatibility and Antifouling Performance of the PLEL Hydrogel

**Figure 3:**
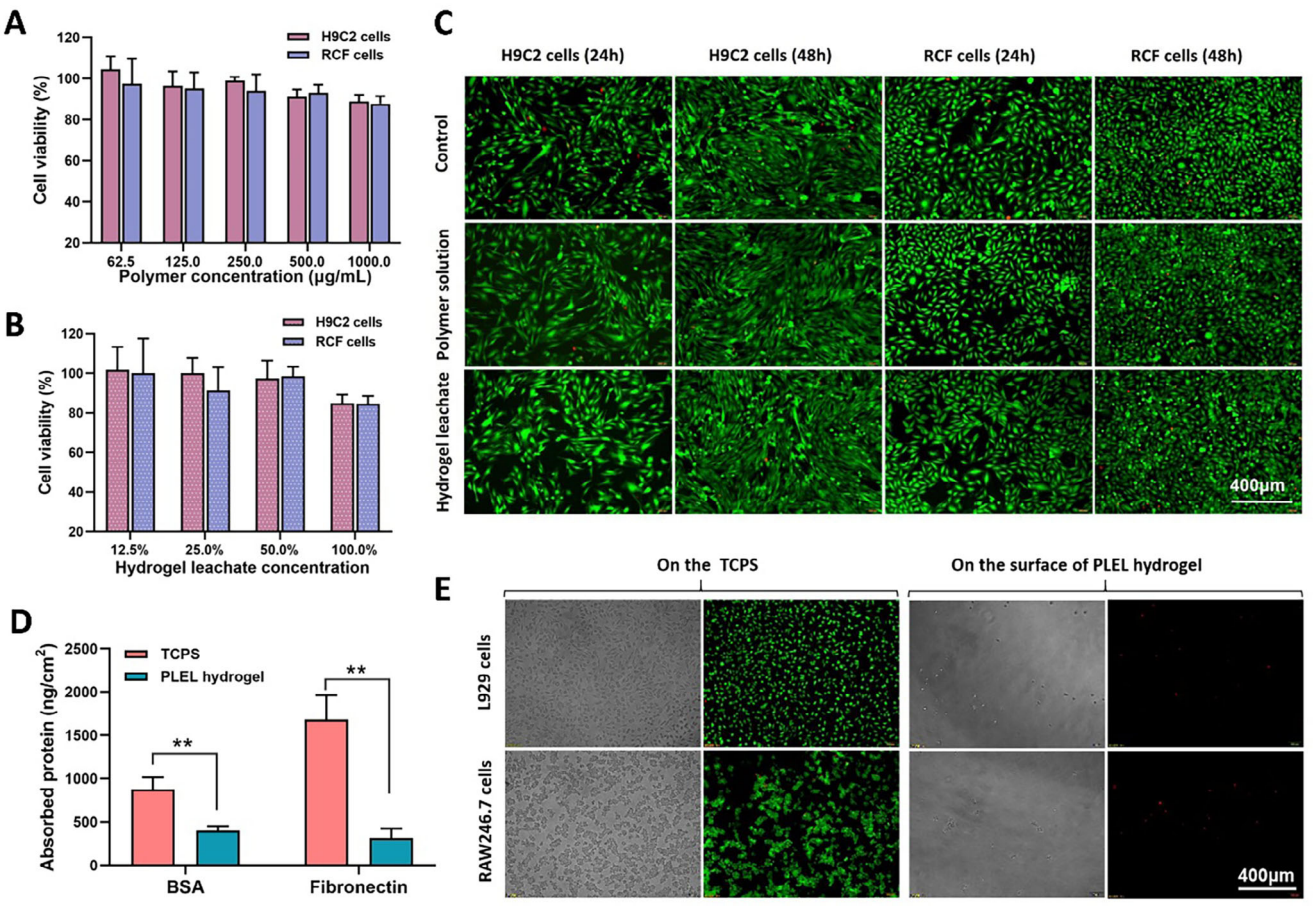
Cytotoxicity, and protein/cell resistance of the PLEL hydrogel. A) Cytotoxicity of the PLEL copolymer against H9C2 cells and RCF cells after coculture for 24 h. Data are presented as the mean ± SD. (*n* = 5). B) Cell viability of H9C2 cells and RCF cells after treatment with PLEL hydrogel leachate for 24 h. Data are presented as the mean ± SD. (*n* = 5). C)Live/dead imaging of the H9C2 cells and RCF cells after treatment with the PLEL polymer or hydrogel leachate for 24 h and 48 h. D) BSA and fibronectin absorption on the surface of the PLEL hydrogel and tissue culture plate surface (TCPS). E) Cellular attachment of L929 cells and RAW246.7 cells on the surface of PLEL hydrogel and TCPS after 24 h, as observed via microscopy and live/dead staining. **p < 0.01.

Myocytes and fibroblasts are the major constituent cells of the heart. As a cardiac anti‐adhesion material, the biocompatibility of PLEL hydrogel with these cells is critical. Thus, rat cardiac myocyte H9C2 cells and cardiac fibroblast RCF were used here to assess the cytocompatibility of the PLEL hydrogel through a cell viability test and live/dead imaging. According to the results in **Figure** **3**A,B; Figure S3 (Supporting Information), neither the PLEL polymer nor the hydrogel leachate exhibited significant cytotoxicity to H9C2 and RCF cells after being co‐cultured for 24 and 48 h. The results of the live/dead assay further confirmed that both the proliferation and morphology of H9C2 and RCF cells were not affected by the PLEL hydrogel, with more than 95% of cells viable and proliferating, indicating acceptable cytocompatibility (Figure 3C; Figures S3C and S3D, Supporting Information).

It has been well‐recognized that fibroblast adhesion and fibronectin adsorption play critical roles in the development of adhesion after operation.^[^ ^32^, ^33^ ^]^ Thus anti‐adhesion barriers with fouling resistance properties are conducive to preventing adhesion formation. As mentioned previously, the PLEL hydrogel was made up of core‐shell‐like micelles with a hydrophobic PDLLA core and a hydrophilic PEG shell.^[^ ^27^, ^30^ ^]^ On account of PEG being a well‐known anti‐cell adhesion material,^[^ ^29^, ^34^ ^]^ we expected that a potential anti‐fouling ability could also be found in our PLEL hydrogel. For this purpose, protein absorption and cell attachment on the surface of the PLEL hydrogel were tested in vitro, and the results are shown in Figure 3D,E. After immersion in the BSA or fibronectin solution for 2 h, the amounts of BSA and fibronectin absorbed on the tissue culture plate surface (TCPS) were measured to be 878 and 1687 ng cm^−2^, respectively. In contrast, the absorption amounts of these two proteins were 399 and 316 ng cm^−2^ on the surface of the PLEL hydrogel, suggesting that the PLEL hydrogel possesses good antiabsorption performance against fibronectin and nonspecific proteins. We further seeded L929 fibroblasts and RAW246.7 macrophages on the surface of the PLEL hydrogel and TCPS to observe their attachment and viability by microscopy after incubation for 24 h. Few spherical cells were found on the surface of the PLEL hydrogel, and almost all of them were dead with positive propidium iodide (PI) staining, whereas many L929 and RAW246.7 cells with viability settled and spread well on the TCPS (Figure 3E). The low protein adsorption and low degree of cell adhesion indicated the favorable anti‐fouling ability of the PLEL hydrogel, which was closely correlated with the hydration of the PEG shell of the PLEL micelles and was likely beneficial for cardiac anti‐adhesion in vivo.

### 2.3 Postoperative Adhesion Prevention Process and Safety Evaluation of the PLEL Hydrogel in a Rat Cardiac Injury Adhesion Model

**Figure 4:**
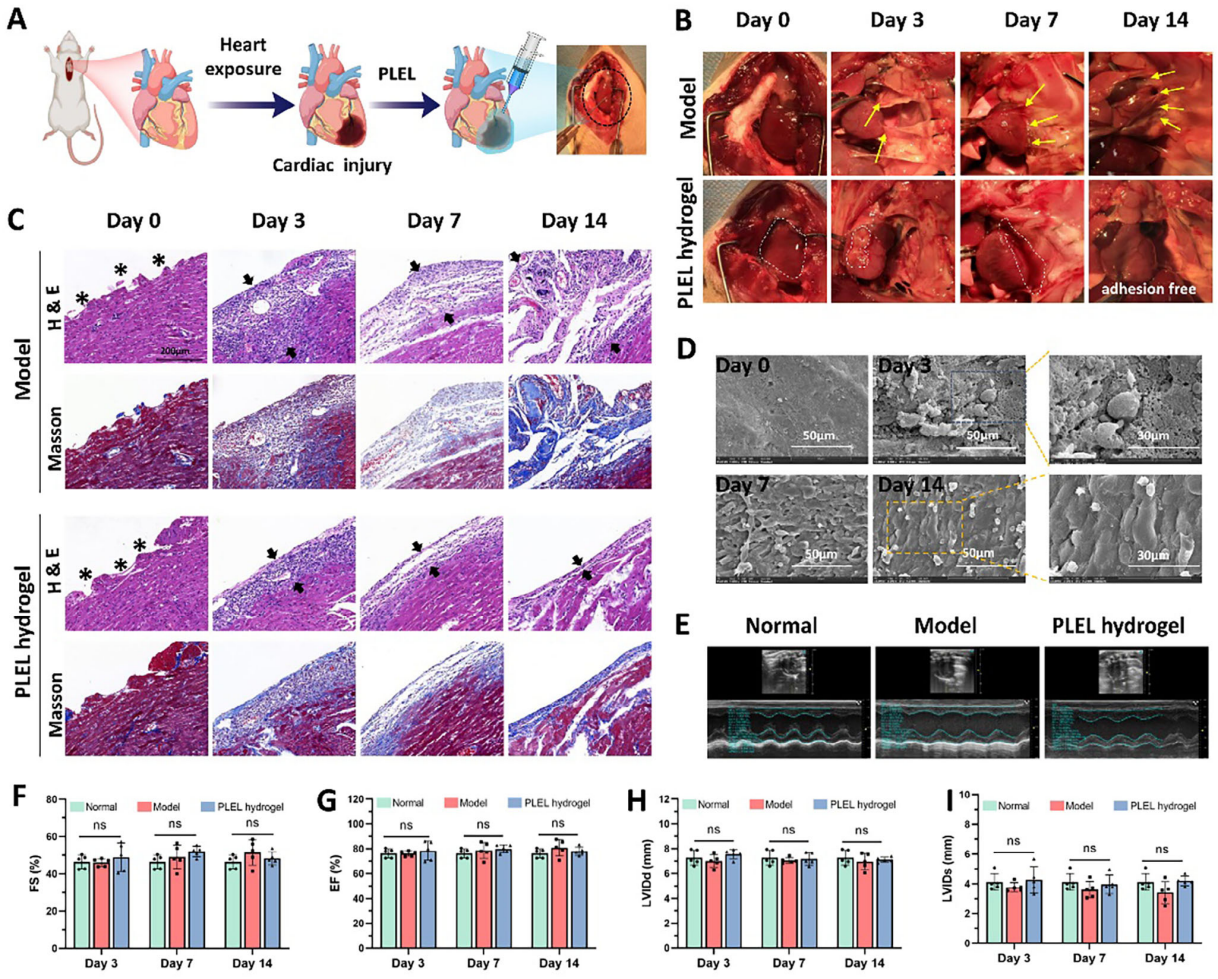
Postoperative adhesion prevention process and safety evaluation of the PLEL hydrogel in a rat cardiac defect adhesion model. A) Schematic diagram of the in vivo anti‐adhesion study using a cardiac adhesion model. B) Representative images of cardiac adhesion in the untreated Model group and the PLEL hydrogel‐treated group on different days after surgery. The yellow arrows indicate the adhesion tissues and the white dashed circles indicate the area of the PLEL hydrogel. C) H&E staining (upper) and Masson's trichrome staining (lower) of samples from Model and PLEL hydrogel groups at different times. The black stars denote the damaged heart surface and the black arrows represent the adhesion/ inflammatory matrix over the wounds. D) Scanning electron microscopy images of the surface of the injured heart after treatment with the PLEL hydrogel. E) Representative images of echocardiographic images for cardiac function evaluation in the treated or untreated group 7 days after cardiac surgery. Normal rats that did not undergo surgery were used as controls. F–I) Measurements of the ejection fraction (EF), fraction shortening (FS), left ventricle internal dimensions at end‐systole (LVIDs), and left ventricle internal dimensions at diastole (LVIDd) on the 3rd, 7th, and 14th days after surgery on the basis of the results of cardiac ultrasound. (*n* = 5). The ns indicates no significant difference.

The cardiac anti‐adhesion efficacy of the PLEL hydrogel was preliminarily studied via an established and highly reproducible cardiac injury adhesion model in rats (**Figure** **4**A).^[^ ^14^, ^31^ ^]^ Figure 4B showed the representative images of rat hearts at different times to study the formation of adhesion and assess the degradation of the hydrogel. On gross examination, almost all the rats in the untreated Model group developed adhesions between the injured heart surface and sternum, and the strength and extent of these adhesions increased with time. On the third day after the operation, a few fragile filmy adhesions (indicated with yellow arrows) appeared, and they were easy to be bluntly separated. The adhesions became stronger and more extensive in the following days, and the hearts were completely adhered to the chest wall on the 14th day with large vascularized fibrous tissues, demonstrating the reliability of this model in the formation of severe cardiac adhesions after surgery. In contrast to those in the Model group, none of the PLEL hydrogel‐treated rats suffered from severe adhesions. After injection, the PLEL hydrogel formed a complete coverage on the surface of the injured heart rapidly, which was still partially visible on the wounds 3 days later (marked via white dashed circles). The hydrogel then transformed into lubricous viscous liquid under the mechanical force of cardiothoracic activity and dilution of the thoracic fluid approximately on the seventh day and was finally absorbed within 14 days. PLEL hydrogel is a polyester‐polyether hydrogel, which can be degraded in vivo by hydrolysis and some enzyme‐assisted biodegradation.^[^ ^27^ ^]^ Importantly, the injuries to the heart and sternum healed gradually and were almost completely recovered by 14 days. These results indicated that the biodegradable PLEL hydrogel was able to cover the defects of the heart reliably and serve as a barrier to prevent the formation of adhesions during the first few postoperative days and then was cleared over time without impeding the recovery of the wounds.

H&E and Masson's trichrome staining was further conducted to study the pathological changes in injured heart tissue during adhesion formation and healing (Figure 4C). Three days after the operation, the untreated cardiac defects were covered by a layer of fibrin matrix with many erythrocytes, macrophages, neutrophils, and other inflammatory cells, while little scattered collagen deposition was observed, suggesting that adhesion formation was closely related to inflammation. The matrix was gradually replaced by vascular granulation tissue containing fibroblasts and macrophages and matured into a fibrous band with abundant collagen deposition on the 14th day.^[^ ^35^ ^]^ After treatment with the PLEL hydrogel, the inflammatory cell matrix on the surface of the damaged heart was significantly thinner than that on the surface of the untreated heart and decreased from the 3rd day till the 14th day, which may be attributed to the fouling‐resistant properties of PLEL hydrogel. The injured tissue began to remesothelialize ≈7 days after the treatment, with fibroblasts, macrophages, and some collagen structures under the spindle neo‐mesothelial cells. Finally, the layer of inflammatory cells was replaced by a thin layer of fibroblasts and collagen‐rich tissue over the next days, indicating complete recovery of the wound. In addition, SEM was also used to observe the morphological changes in the remesothelialization of cardiac injuries after being treated with PLEL hydrogel. As shown in Figure 4D, the mesothelium and even the myocardium were damaged by surgical trauma on day 0. Three days after treatment, there were still some hydrogel materials covered on the meshwork of fibrin, while erythrocytes and spherical inflammatory cells were observed in the higher‐magnification image. On the 7th day, most of the injured tissue was re‐covered by elongated, squamous‐like mesothelial cells, which overlapped loosely with each other. Another 7 days later, the mesothelial cells became tight and completely overlaid the damaged sites, demonstrating full regeneration of the cardiac injury.^[^ ^36^ ^]^

As mentioned previously, because of its 400% swelling, the CoSeal hydrogel has been reported to cause cardiac tamponade in several patients in clinical trials when serving as an adhesion barrier, resulting in compression of the heart and cardiac dysfunction.^[^ ^14^, ^24^ ^]^ Therefore, we assessed the cardiac function of rats after treatment with the PLEL hydrogel using M‐mode echocardiography. The results of echocardiography on days 3, 7, and 14 after the abrasion and hydrogel application did not show any signs of cardiac dysfunction (Figure 4E–I). There was no significant difference in the ejection fraction (EF), fraction shortening (FS), left ventricle internal dimensions at end‐systole (LVIDs) and left ventricle internal dimensions at diastole (LVIDd) between all the groups at different postoperative times. Rats treated with the PLEL hydrogel were closely monitored and none of them displayed any adverse reactions such as abnormal food or water intake, slow movement, or eye secretion.^[^ ^10^ ^]^ Meanwhile, H&E staining of the vital organs (heart, liver, spleen, lung, and kidney) after surgery and treatment was also conducted to evaluate the side effects. Figure S4 (Supporting Information) showed that almost all the tissues of the rats treated with the PLEL hydrogel exhibited comparable histological morphologies to those of the rats in the Model and Normal group. According to the blood chemistry tests, no substantial abnormalities were observed in the PLEL hydrogel‐treated group either (Figure S5, Supporting Information). All of these results demonstrated the safety of the PLEL hydrogel when it was used in cardiac surgery for adhesion prevention.

### 2.4 PLEL Hydrogel Prevented Postoperative Adhesion in a Rat Cardiac Injury Adhesion Model

**Figure 5:**
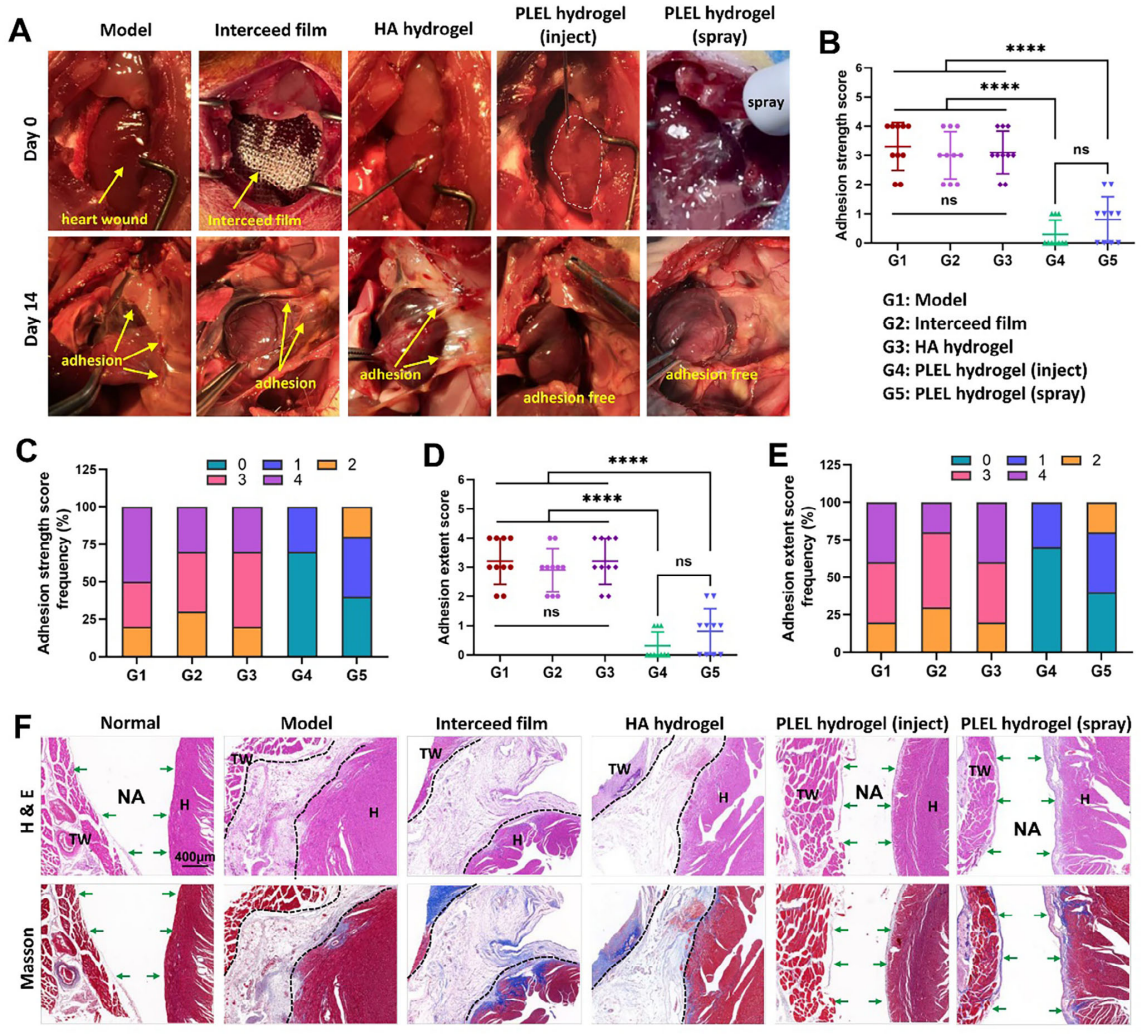
Anti‐adhesion efficiency of the PLEL hydrogel in a rat cardiac injury adhesion model. A) Representative photographs of treatments during surgery and cardiac adhesion on postoperative day 14 in the Model, Interceed film, HA hydrogel, injected PLEL hydrogel and sprayed PLEL hydrogel groups. The yellow arrows indicate the adhesion tissues. B) Standard adhesion scoring system on a scale from 0 to 4. C–F) The adhesion strength/extent score and their corresponding frequency results of the different groups after treatment on postoperative day 14 (*n* = 10). G) H&E staining and Masson trichrome staining of injured heart and thoracic wall tissues from different groups 14 days after surgery (H: heart; TW: thoracic wall; NA: no adhesion; dashed area: adhesion tissue; green arrows: intact mesothelial layer). The ns indicates no significant difference. ****p < 0.0001.

Encouraged by the aforementioned positive results of the PLEL hydrogel in preventing cardiac adhesion and in terms of its safety, we further evaluated its anti‐adhesion efficiency in a rat model of cardiac injury adhesion in detail via a commonly used adhesion scoring system in comparison with commercial anti‐adhesion film and hydrogel products. After an injury to the heart was induced, the PLEL hydrogel was injected through a syringe (denoted as the PLEL hydrogel (inject)) or sprayed via a medical throat sprayer (denoted as the PLEL hydrogel (spray)) around the surfaces of the wound areas, followed by a rapid and spontaneous gelation at the site (**Figure** **5**A). As a positive control, the defects were covered by the commercial Interceed film or sodium hyaluronate (HA) hydrogel. For negative model control, the injured area was washed only with normal saline carefully without other treatments.

Fourteen days after surgery, a gross assessment of cardiac adhesion formation was performed by reopening the chest through a lateral thoracotomy after the rats were euthanized. The representative images of the adhesions in each group are shown in Figure 5A, and all groups were scored in a double‐blind manner according to a standard adhesion scoring system on a scale from 0 to 4 (**Table** **1**).^[^ ^7^, ^14^ ^]^ As shown in Figures 5B‐E, all rats in the Model group suffered from severe adhesions between the heart and sternum, with an average strength score of 3.3 and an average extent score of 3.2. After treatment with the commercial Interceed film or HA hydrogel, although the adhesions were alleviated to some extent, the adhesion scores were not significantly decreased (mostly scored greater than 3) when compared with the untreated group. By contrast, both the injected PLEL hydrogel and the sprayed PLEL hydrogel tremendously reduced the adhesion strength and extent. In the injected PLEL hydrogel treatment group, seven out of ten did not develop any adhesion (score 0), and three out of ten presented slight adhesion (score 1), resulting in an average score as low as 0.3 for both adhesion strength and adhesion extent. There was no statistical difference between the injected PLEL hydrogel and sprayed PLEL hydrogel treatment groups (*p *> 0.05), suggesting that the administration of the PLEL hydrogel can be flexibly selected according to the actual needs of different surgeries for adhesion prevention. The change in body weight after surgery and treatment didn't significantly differ among these five groups (Figure S6, Supporting Information).

**Table 1:** Cardiac adhesion scoring criteria.

Score	Adhesion Strength	Adhesion Extent
0	No adhesions	No adhesions
1	Weak filmy adhesions, blunt dissection	Single band of adhesion
2	Moderately strong adhesions, partly sharp dissection	Two bands of adhesions
3	Strong adhesions, sharp dissection	More than two bands
4	Strong vascularized adhesions, sharp dissection	Multiple dense adhesions

**Figure 6:**
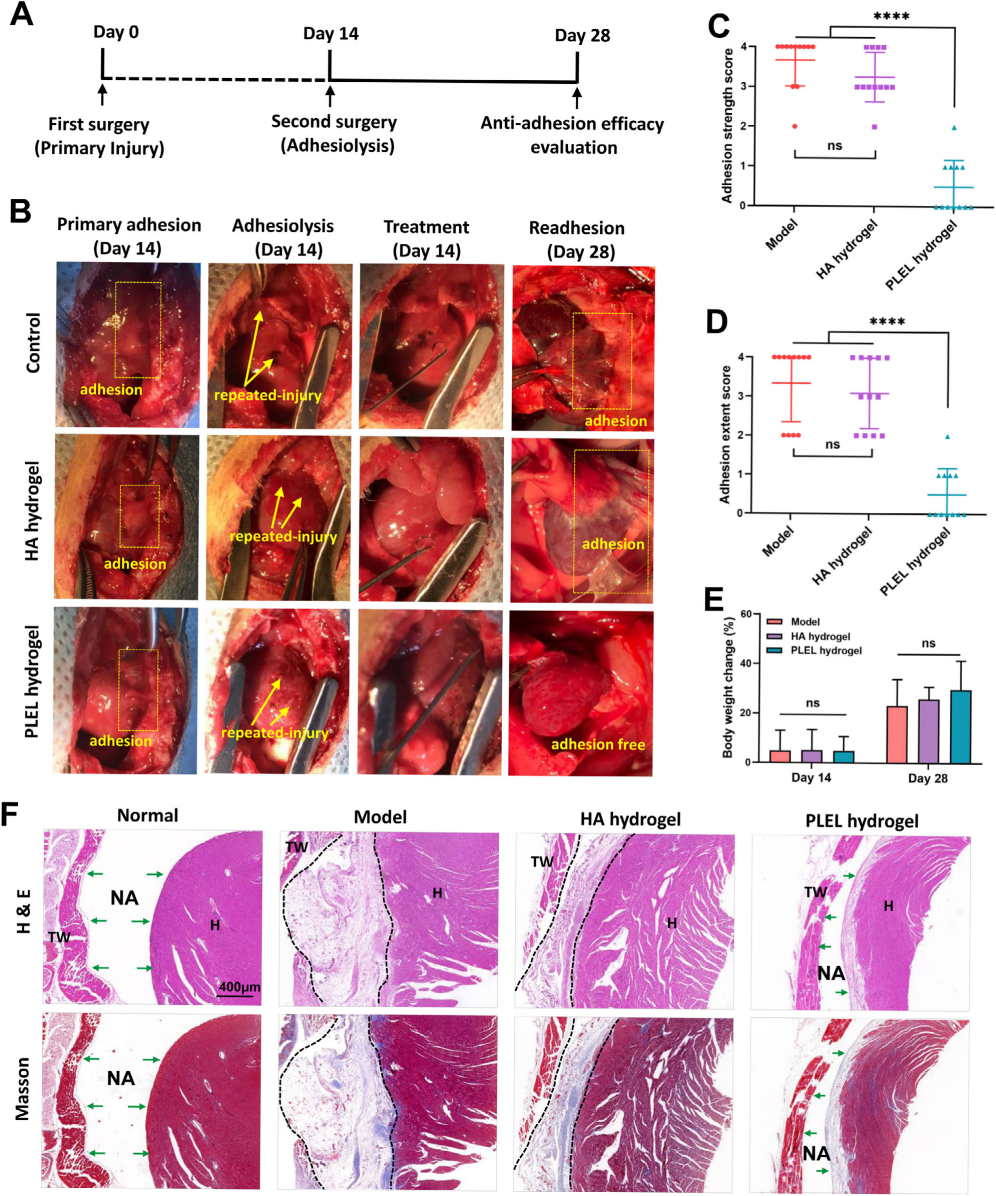
Evaluation of recurrent adhesion prevention in a rat repeated‐injury cardiac adhesion model. A) Schematic of the experimental schedule. B) Representative photographs of primary cardiac adhesion, wounds, and treatment after adhesiolysis, and recurrent cardiac adhesions on postoperative day 28 in the Model, HA hydrogel, and injected PLEL hydrogel groups. The yellow dotted rectangles denote the area of adhesion tissue and the yellow arrows indicate the repeated injury after adhesiolysis. C, D) The adhesion strength/extent score of different groups after treatment on day 14 after adhesiolysis (*n* = 12). E) Changes in the body weight of the rats in the different groups 14 days after the first surgery and adhesiolysis (*n* = 12). F) H&E staining and Masson trichrome staining of injured heart and thoracic wall tissues from different groups 14 days after adhesiolysis (H: heart; TW: thoracic wall; NA: no adhesion; dashed area: adhesion tissue; green arrows: intact mesothelial layer). The ns indicates no significant difference. ****p < 0.0001.

Tissue samples from the injured sites in the different groups were collected, and H&E staining and Masson's trichrome staining were performed to histologically visualize tissue adhesion and heart healing after cardiac surgery. As exhibited in Figure 5F, the chest wall and the injured heart were fused with accumulated connective tissues (marked by the dashed line) in the untreated Model group, accompanied by a mass of fibroblasts, inflammatory cells, collagen depositions (stained with intense blue in Masson staining), and new blood vessels, indicating the formation of mature adhesions. Consistent with the gross examination, similar adhesion tissues were also found in the commercial Interceed film or HA hydrogel treatment groups, whereas no apparent adhesion was formed after treatment with the PLEL hydrogel. It was clearly observed that the injured surfaces of the hearts in the PLEL group were recovered with a fully developed mesothelium layer (marked by green arrows) with some collagen deposition, which was similar to that in the normal tissue, demonstrating good anti‐adhesion effects without interfering with the regular wound healing.^[^ ^10^, ^18^ ^]^

### 2.5 PLEL Hydrogel Prevented Recurrent Adhesion After Adhesiolysis in a Rat Repeated‐Injury Cardiac Adhesion Model

In clinical practice, secondary surgery or staged operations are in high demand for patients with heart conditions, and adhesiolysis is inevitable to release the preexisting postoperative adhesions before the reoperation. Unfortunately, the new trauma caused by surgical lysis tends to trigger a high incidence of recurrent adhesion (more than 55%) even if minimally invasive procedures are used.^[^ ^37^, ^38^ ^]^ Because recurrent adhesions are more complicated and severe, they are more difficult to prevent than primary adhesions. In fact, many clinical treatments and antiadhesion materials under development have focused on primary cardiac adhesion prevention, while few have targeted recurrent adhesion prevention after adhesiolysis or have shown sufficient efficacy.^[^ ^12^, ^28^ ^]^ Therefore, we further evaluated the anti‐adhesion efficacy of PLEL hydrogel in a more rigorous rat repeated‐injury recurrent cardiac adhesion model that was much closer to that in clinical practice. We considered the fact that the HA hydrogel and Interceed film showed a similar effect in anti‐adhesion in the primary adhesion model, and there were no statistical differences between the Injected PLEL hydrogel treated group and the Sprayed PLEL hydrogel treated group as well. Therefore, we further evaluated the anti‐adhesion efficiency of injected PLEL hydrogel in a more serious repeated‐injury adhesion model, using the HA hydrogel as a positive group and the untreated model group as a negative group. As shown in **Figure** **6A,B**, the repeated‐injury cardiac adhesion model was established by creating a primary cardiac abrasion without any treatment, followed by a second thoracotomy on day 14 to lyse the primary adhesion between the heart and the adjacent tissues, and then abrading the surface of the separated heart again to produce secondary injuries (marked by yellow arrows). Before the final closure, the repeated‐injured tissues in the treatment groups were covered with either the PLEL hydrogel or the commercial HA hydrogel, and the recurrent adhesions were checked (Figure 6B) and scored (Figure 6C,D) on the 28th day.

In the untreated Model group, the injured heart was completely adhered to the chest wall with multiple dense vascularized adhesions, with an average strength score of 3.7 and an average extent score of 3.3. Adhesions were not only visible between the injured heart and sternum but also involved the adjacent lung, and the adhesion area was larger than that of the initial wound, indicating complicated and severe recurrent adhesions. In the group treated with the HA hydrogel, most of the rats still suffered recurrent adhesions, similar to those in the Model group, but both the strength and extent of adhesions were reduced slightly. In contrast, the use of the PLEL hydrogel significantly reduced the incidence and severity of recurrent adhesions, with adhesion scores of 0.5, demonstrating the excellent preventive effect of the PLEL hydrogel on recurrent adhesion after adhesiolysis. The change in body weight during the treatment also did not differ significantly among the groups (Figure 6E). The results from H&E and Masson staining supported the gross scoring: both the untreated and HA hydrogel‐treated groups presented distinct mature adhesions between the injured heart and chest wall with more inflammatory cells, greater neovascularization, and denser collagen fiber deposition (dashed area), whereas the injured sites of the majority of the rats in the PLEL hydrogel group completely recovered without obvious adhesion tissues or observable infiltrated inflammatory cells (Figure 6F). These results showed that the PLEL hydrogel can effectively prevent cardiac recurrent adhesion after adhesiolysis, suggesting that it has a high potential to address this difficult clinical issue in cardiac surgery.

### 2.6 Effects of PLEL Hydrogel on the Inflammatory Response and Fibrinolytic System

Previous studies have shown that the formation of postoperative adhesion involves a cascade of coordinated events, such as trauma, inflammation, wound healing, fibrosis, and fibrinolysis. Specifically, the inevitable inflammatory response triggered by the trauma promotes the release of a number of cytokines, accelerates the migration of monocytes and macrophages, and increases the effusion of fibrin‐rich exudates, leading to the formation of fibrinous adhesion. If plasminogen‐plasmin cascade‐mediated fibrinolysis is not active enough, fibrinous adhesions are invaded by fibroblasts and accompanied by the deposition of collagen, resulting in the development of dense and vascularized fibrous adhesions.^[^ ^9^ ^]^ Thus, inflammation‐related immune reactions and a disrupted fibrinolytic system after injury are considered to be the main reasons for the development of tissue adhesions.^[^ ^18^, ^28^, ^39^ ^]^ To further investigate the potential anti‐cardiac adhesion mechanism of the PLEL hydrogel, we performed immunofluorescence staining and ELISA to evaluate the effects of the PLEL hydrogel on the inflammatory response and fibrinolytic system in a cardiac injury adhesion model and a repeated‐injury adhesion model.

**Figure 7:**
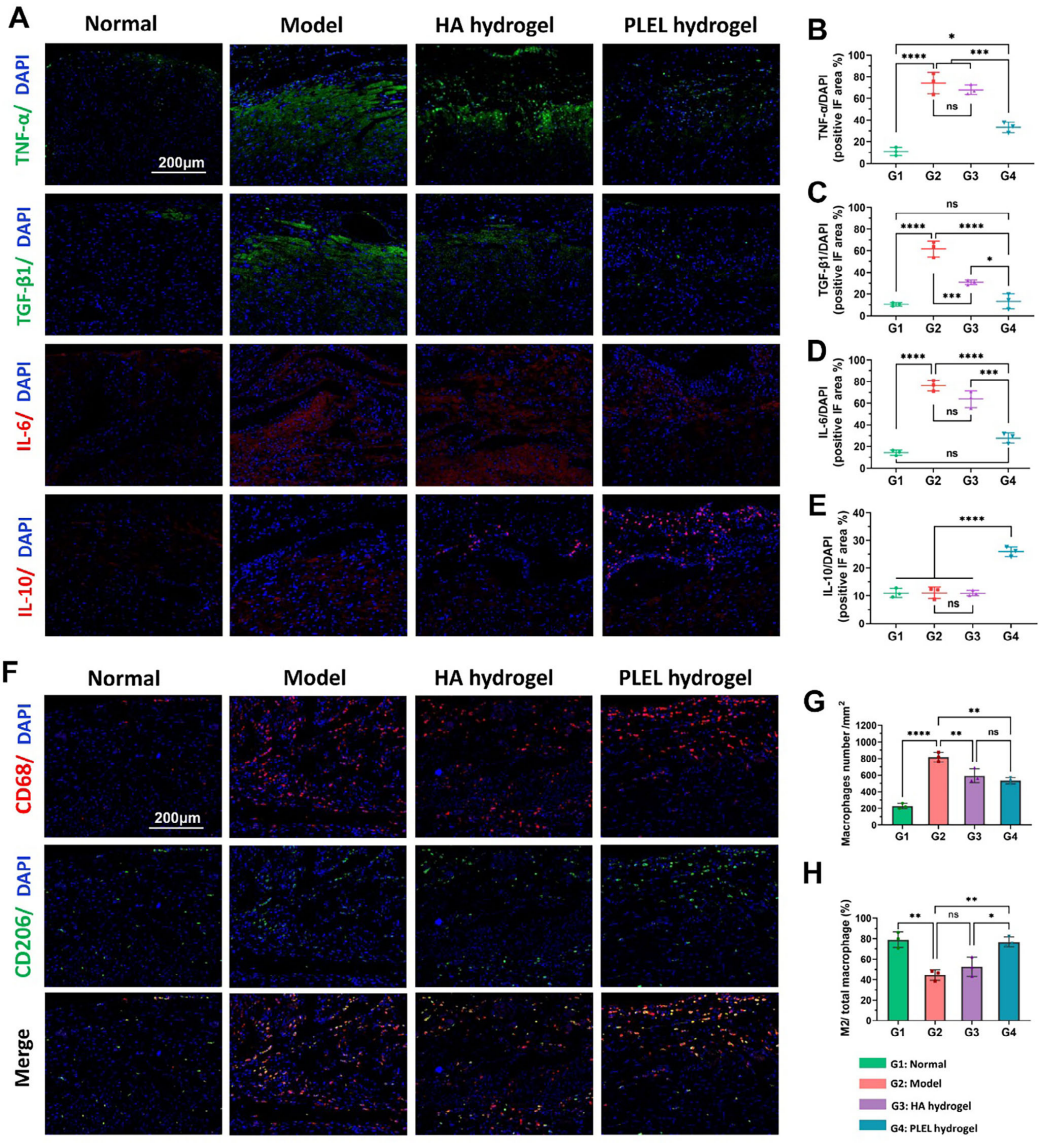
Effects of the PLEL hydrogel on the inflammatory response in a rat model of cardiac injury adhesion. A) IF staining images of TNF‐α (green), TGF‐β1 (green), IL‐6 (red), IL‐10 (red), and DAPI (blue) in heart and adhesion tissues from different groups two weeks after surgery. B–E) Fluorescent area ratios of TNF‐α/DAPI, TGF‐β1/DAPI, IL‐6/DAPI, and IL‐10/DAPI (*n* = 3). F) IF staining images of CD68 (red), CD206 (green), and DAPI (blue) in heart and adhesion tissues from different groups two weeks after surgery. The expression of CD68 represents total macrophages, and that of CD206 represents M2 macrophages. G) The number of total macrophages obtained by counting the number of CD68‐positive cells (*n* = 3). H) The ratio of M2 macrophages labeled with CD206 to total macrophages (*n* = 3). The ns indicates no significant difference. *p < 0.05, **p < 0.01 and ***p < 0.001, ****p < 0.0001.

**Figure** **7**A–E showed the expression levels of proinflammatory cytokines such as tumor necrosis factor‐α (TNF‐α), transforming growth factor‐β1 (TGF‐β1), interleukin‐6 (IL‐6), and anti‐inflammatory cytokine interleukin‐10 (IL‐10) in the primary injured cardiac tissue of each group at two weeks after surgery. TNF‐α is regarded as the most important inflammatory factor in the formation of adhesion, which not only promotes the inflammatory response but also disrupts the fibrinolytic system by inhibiting the activity of tissue‐type plasminogen activator (t‐PA) and stimulating the release of its inhibitors (such as plasminogen activator inhibitor‐1 (PAI‐1)).^[^ ^28^ ^]^ Compared with the normal heart tissue, surgical trauma in the model group significantly increased the expression of TNF‐α. Compared with the Model group, the TNF‐α expression in the commercial HA hydrogel‐treated group was only slightly reduced, without statistical difference. In contrast, the PLEL hydrogel had the most pronounced effect on inhibiting the expression of TNF‐α. TGF‐β1 and IL‐6 participate in the inflammatory reaction by facilitating the release of cytokines, recruiting inflammatory mediators to the wound, and accelerating fibrin formation and tissue fibrosis, which significantly impacts the formation of adhesions.^[^ ^40^, ^41^ ^]^ The results of immunofluorescence staining and quantitative analysis revealed that the expression of TGF‐β1 and IL‐6 in the Model group was notably greater than that in the normal group. After treatment with the PLEL hydrogel, the levels of both TGF‐β1 and IL‐6 were reduced to levels that were not significantly different from those in the normal group (*p *> 0.05). We also found that the use of the PLEL hydrogel markedly promoted the expression of the anti‐inflammatory cytokine interleukin‐10 (IL‐10), which contributed to the inhibition of adhesion and the facilitation of wound healing. Moreover, the changes in these inflammatory cytokines after different treatments in the repeated‐injury recurrent cardiac adhesion rat model were also determined in detail and are presented in the supporting information (Figure S7A–E, Supporting Information), where the observed tendencies were similar to those in the first injured rat cardiac adhesion model.

The aforementioned H&E staining of injured heart tissue (Figures 5, and 6) showed that a large number of inflammatory cells infiltrated the injured site and adhesion tissue, including macrophages, neutrophils, monocytes, etc. Among them, macrophages play an important role in mediating the inflammatory response. In general, a more severe inflammatory response is often accompanied by increased infiltration of macrophages, most of which show pro‐inflammatory effects, and only a small number of M2‐type macrophages have anti‐inflammatory effects and participate in tissue repair.^[^ ^42^ ^]^ Therefore, macrophage infiltration and M2 macrophage regulation in the tissues collected from each treatment group were assessed by immunofluorescence. CD68 and CD206 are crucial markers of total macrophages and M2 macrophages, respectively.^[^ ^43^ ^]^ As shown in Figure 7F–H, 14 days after the cardiac injury adhesion model was established, a number of macrophages were recruited to the injury site and adhesion area in the model group, 44.5% of which were M2 macrophages. Compared with those in the Model group, the number of macrophages was significantly lower after treatment with the HA hydrogel or PLEL hydrogel, especially in the PLEL hydrogel group, which may benefit from its fouling resistance. It should be noted that the proportion of M2 macrophages among the total macrophages was as high as 76.8% in the PLEL hydrogel‐treated group, indicating a better effect on M2 macrophage polarization. In the case of the repeated‐injury recurrent cardiac adhesion model, the minimum macrophage infiltration and the highest M2 macrophage ratio were also found in the PLEL hydrogel treated group, whereas those of the HA hydrogel group were not significantly different from those of the Model group (Figure S7F–H, Supporting Information). These results suggested that the PLEL hydrogel can dramatically alleviate the injury‐induced inflammatory response by inhibiting pro‐inflammatory cytokines release and macrophage infiltration and increasing the levels of anti‐inflammatory factors and ratio of M2 macrophages.

**Figure 8:**
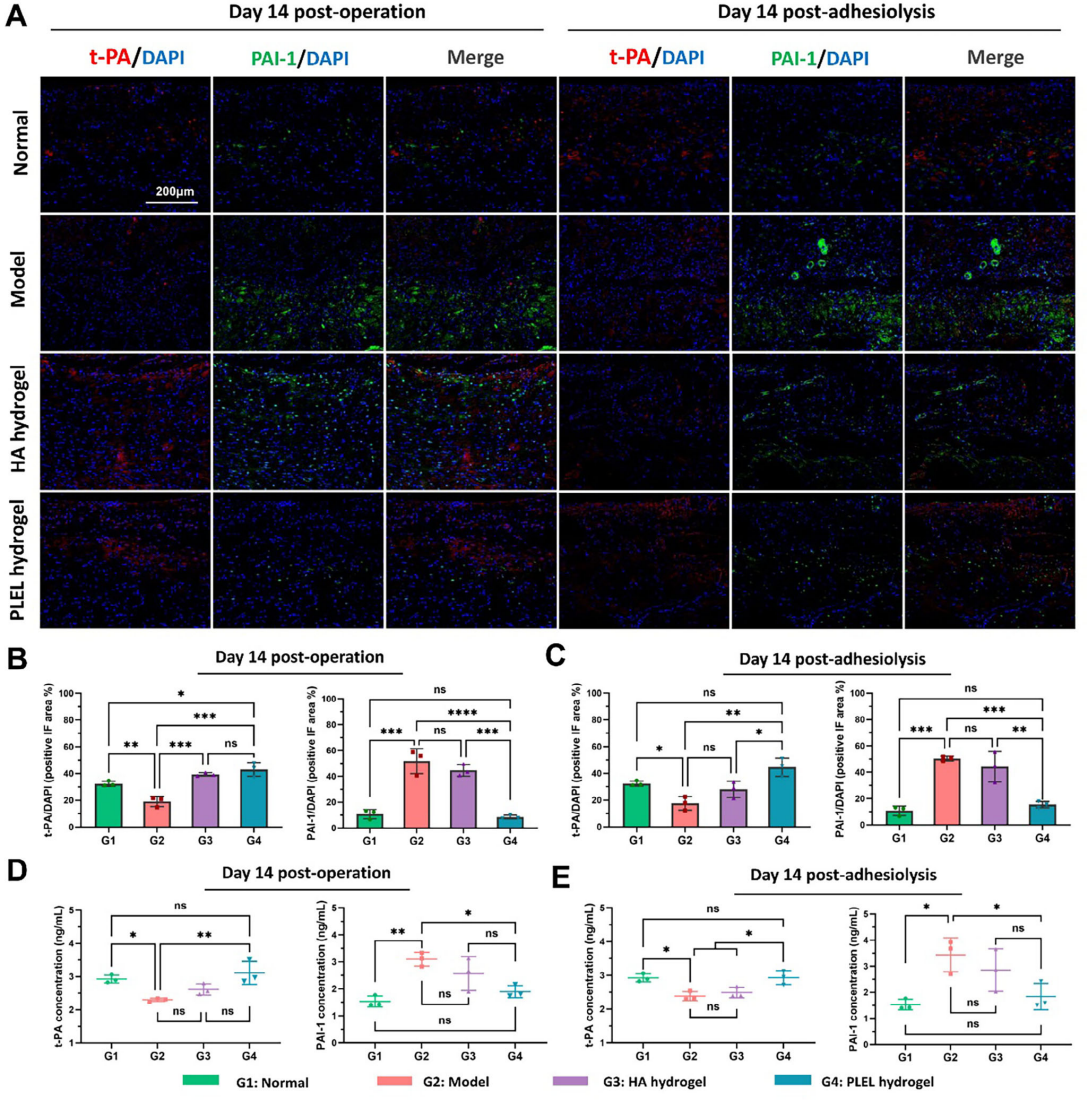
Effects of the PLEL hydrogel on the fibrinolytic system in a rat cardiac injury adhesion model and a repeated‐injury cardiac adhesion model. A) IF staining images of t‐PA (red), PAI‐1 (green), and DAPI (blue) in heart and adhesion tissues from the different groups on day 14 after primary surgery and day 14 after adhesiolysis. B, C) Fluorescent area ratios of t‐PA/DAPI and PAI‐1/DAPI in different groups on day 14 after primary surgery and day 14 after adhesiolysis. (*n* = 3). D, E) Concentrations of t‐PA and PAI‐1 in the serum on day 14 after primary surgery and day 14 after adhesiolysis (*n* = 3). The ns indicates no significant difference. *p < 0.05, **p < 0.01 and ***p < 0.001, ****p < 0.0001.

Additionally, the effect of the PLEL hydrogel on the fibrinolytic system of surgical wounds was investigated by immunofluorescence (IF) staining and ELISA. Tissue‐type plasminogen activator (t‐PA) and plasminogen activator inhibitor‐1 (PAI‐1) are important components of the fibrinolytic system that work by regulating fibrin deposition and fibrinolysis. After injury, the balance between t‐PA and PAI‐1 is disrupted by the inflammatory response and excessive coagulation, which is closely related to the formation of postoperative adhesions.^[^ ^44^ ^]^ **Figure** **8**A–C showed the immunofluorescence staining images and analysis results of t‐PA and PAI‐1 in the injured cardiac tissue of each group on the 14th day after the first operation and adhesiolysis. In the model group, surgical trauma resulted in a low expression of t‐PA (red fluorescence) and a high expression of PAI‐1 (green fluorescence). After treatment with the HA hydrogel, the expression of t‐PA increased to a certain extent, while the expression of PAI‐1 did not significantly differ from that in the Model group (*p *> 0.05). However, in the PLEL hydrogel‐treated group, obvious positive IF areas of t‐PA but few PAI‐1 expressing areas were observed in both the first surgical model and the repeated injured model, which were similar to those of the normal group. Moreover, the corresponding t‐PA and PAI‐1 levels in the serum were consistent with the IF staining results above (Figure 8D,E). Therefore, we concluded that the PLEL hydrogel could not only effectively promote the expression of t‐PA but also weaken the expression of PAI‐1 after surgery, suggesting the potential ability to balance the fibrinolytic system and prevent cardiac adhesion.

## 3 Conclusion

In summary, a thermosensitive in‐situ forming hydrogel with rapid gelation and anti‐fouling performance was developed to prevent primary and more severe recurrent cardiac post‐operative adhesion. Its injectability and sprayability make it possible to cover large and irregular wound areas uniformly by spraying or injecting them to the injured site through minimally invasive thoracoscopy and catheters, providing choices for different surgical interventions. After being subjected to heart trauma, stable hydrogel coverage could form immediately in response to body temperature and then serve as a barrier between the wounds during the main period of adhesion formation without the risk of affecting cardiac function or causing tissue toxicity. Compared with commercial anti‐adhesion film and hydrogel products, this PLEL hydrogel has been demonstrated to significantly reduce the incidence and severity of postoperative cardiac adhesions in rats and recurrent adhesions after adhesiolysis. In addition to being an effective physical barrier between wounds, several important functions of PLEL hydrogel are potentially involved in the anti‐adhesion mechanism, including resisting protein adhesion and cell invasion during the trauma, inhibiting the inflammatory response, and balancing the fibrinolytic system. Considering its simple preparation process, ease of operation, suitable residence time, excellent biocompatibility, and powerful effects on postoperative and recurrent cardiac adhesion prevention, this thermosensitive PLEL hydrogel is believed to be a highly promising anti‐adhesion material to meet current clinical needs. Of course, we need to point out that the cardiac post‐operative adhesion prevention efficacy of the PLEL hydrogel needs to be further evaluated in large animal models, such as sheep and pigs, because there are significant differences between small animals and large animals. It is better to evaluate the anti‐adhesion effect in large animal models that can more realistically simulate clinical cardiac surgery, such as the myocardial infarction model and cardiopulmonary bypass and aortotomy model. What's more, we also need to optimize the dosage of PLEL hydrogel and evaluate its long‐term safety in a larger animal model in the future.

## 4 Experimental Section

### Materials

Poly(ethylene glycol) (PEG, *Mn* = 1500) was obtained from Nanjing Well Pharmaceutical Co., Ltd. (China). D,L‐lactide (D,L‐LA) was purchased from Jinan Daigang Biomaterial Co., Ltd. (China). Stannous octoate (Sn(Oct)_2_) and 3‐(4,5‐dimethylthiazol‐2‐yl)‐2,5‐diphenyltetrazolium bromide (MTT) were purchased from Sigm‐Aldrich (USA). The Live & Dead Cytotoxicity Assay Kit was purchased from Jiangsu KGI Biotechnology Company (China). RPMI 1640 medium, Dulbecco's modified Eagle's medium (DMEM), trypsin, fetal bovine serum (FBS), and penicillin‐streptomycin liquid were obtained from Gibco (USA). Commercially available Interceed Absorbable Adhesion Barrier was obtained from Johnson & Johnson. Sodium hyaluronate (HA) antiadhesion hydrogel was obtained from Jingjia Medical (China). Micro BCA protein assay kit was purchased from Sangon Biotech (China). Fibronectin and bovine serum albumin (BSA) were obtained from Beijing Solarbio Science & Technology Co., Ltd. (China). ELISA kits were supplied by Abcam (UK). The other chemical reagents were of analytical pure grade and were used as received.

### Animals

Female Sprague–Dawley rats (SD, 220 ± 20 g) were purchased from Chengdu DOSSY Experimental Animals Co., Ltd. (Chengdu, China) and maintained in a controlled environment with a 12 h light/dark cycle. The animals were provided with free access to food and water. All animal procedures were approved by the Institutional Animal Care and Use Committee (IACUC) of West China Hospital (Checking number: 20220307019) and carried out in accordance with the approved guidelines.

### Preparation and Characterization of the PLEL Hydrogel

PDLLA‐PEG‐PDLLA (PLEL) with a molecular weight of 4500 Da was synthesized by ring‐opening copolymerization as previously described and the methods are reproduced here.^[^ ^30^ ^]^ PEG (10 g) was introduced into a dry flask and heated under vacuum at 100 °C for 1 h to eliminate trace amounts of water. D, L‐lactide (20 g), and stannous octoate (0.09 g) were added under a nitrogen atmosphere, and the mixture was then stirred at 140 °C for 12 h under the protection of nitrogen. The crude copolymer was fully dissolved in water at room temperature, followed by precipitation at 80 °C. The PLEL was collected and dried under a vacuum before being stored at −20 °C. Nuclear magnetic resonance spectroscopy (^1^H‐NMR, Varian 400 spectrometer, Varian, USA) and gel permeation chromatography (GPC, Agilent 110 HPLC, USA) were used to characterize the obtained PLEL copolymer. The morphology of PLEL micelles (1 wt.%) was observed under a transmission electron microscope (TEM, H‐600, Hitachi, Japan) and dynamic light scattering (DLS, Nano‐ZS 90, Malvern, Worcestershire, UK). The PLEL hydrogel was prepared by dissolving the PLEL copolymer in phosphate‐buffered saline (20 wt.%) at room temperature with mild agitation and sterilized with a 0.22 µm filter membrane. The injectability and sprayability of the PLEL solution were tested via injection through a 1 mL 22G syringe or spraying via a 5 mL medical throat spraying bottle at room temperature. The sol‐gel phase transition was recorded after the PLEL solution was injected or sprayed on the surface of a bottle filled with 37 °C water. The in‐situ gelation behavior of the PLEL solution after it was sprayed on the surface of a rat heart was also studied in vivo. To make these performances more perceptible, a water‐soluble blue dye was dissolved in the PLEL solution sample. The swelling of the PLEL hydrogel was recorded by quantifying the wet weight after phosphate‐buffered saline (PBS) incubation at 37 °C. The swelling ratio was calculated using the following formula: swelling ratio = (M_2_‐M_1_)/M1 × 100%, where M_1_ is the original mass of the hydrogel and M_2_ is the swollen mass of the hydrogel after incubation.^[^ ^15^ ^]^

### Rheological Measurements

Dynamic rheological study of PLEL hydrogel was carried out on a rheometer (HAAKE Rheostress 6000, Thermo Scientific, USA) equipped with a parallel plate (diameter: 20 mm). Low‐viscosity silicone oil was used to minimize the water evaporation from the samples during all the measurements. An angular frequency of 10 rad s^−1^ and a 1% strain were selected to ensure that the oscillatory deformation was within the linear regime. Changes in the storage modulus (G′) and loss modulus (G“) were measured as functions of temperature from 10 to 50 °C at a heating rate of 1 °C min^−1^. Time sweep tests were performed at a 1% strain over a time range from 0 to 300 s to detect the gelation time of the PLEL hydrogel at 37 °C. The gelation time was recorded when G' became greater than G”. Frequency sweep tests were performed at a 1% strain over the frequency range of 0.1–100 rad s^−1^ at 37 °C. Strain sweep tests were also carried out at 10 rad s^−1^ over the strain range from 0.1% to 100%. All the rheological properties were analyzed with the instrument software.

### In Vitro Cytotoxicity and Live/Dead Assay

Rat cardiac H9C2 myocytes and rat cardiac fibroblasts RCF were used to examine the in vitro cytotoxicity of the PLEL hydrogel via the MTT assay. H9C2 and RCF cells were purchased from BeNa Culture Collection (China) and cultured in DMEM medium containing 10% FBS, supplemented with 1% penicillin and streptomycin at 37 °C in a humidified 5% CO_2_ atmosphere. PLEL polymer solutions with different concentrations were prepared in DMEM, and the leachate of the PLEL hydrogel was prepared by extraction in DMEM medium for 24 h and sequentially diluted with DMEM. The cell suspension was seeded in 96‐well plates at a density of 3500 cells/well and incubated for 24 h. The medium was then replaced with fresh medium with different concentrations of the PLEL polymer or PLEL hydrogel leachates. After being cultured for another 24 or 48 h, the cells were subjected to the MTT assay, and the absorbance was measured at 570 nm using a microplate reader (Bio‐Rad). The cells cultured with DMEM were used as a negative control for 100% cell viability. For the live/dead assay, H9C2 and RCF cells were cultured in 24‐well plates (3 × 10^4^ cells/well) for 24 h, after which the medium was replaced with PLEL polymer (1 mg mL^−1^) or PLEL hydrogel leachate (100%). After 24 or 48 h of culture, the cells were stained with 2 µM calcein AM and 8 µM PI (Live/Dead cell staining kit) for 30 min at room temperature. The fluorescent images were then observed under a fluorescence microscope (Olympus, Tokyo, Japan), and the images were analyzed via ImageJ 7.0 software.

### Measurement of Protein Adsorption

Fibronectin and nonspecific protein adsorption on disks or PLEL hydrogels was evaluated by using a Micro BCA protein assay kit.^[^ ^12^ ^]^ One milliliter of PLEL solution was added to a 24‐well plate and maintained at 37 °C for 10 min to form a stable hydrogel disk. Then, 0.5 mL of fibronectin or BSA solution (50 µg mL^−1^) was added to cover the hydrogel disks or the tissue culture plate surface (TCPS). After incubation for 2 h at 37 °C, the protein mixture was removed, and the hydrogel or TCPS was carefully rinsed with fresh PBS three times to remove the loosely adsorbed protein. The hydrogel and TCPS were subsequently soaked in 0.5 mL of 1% sodium dodecyl sulfate (SDS) for 1 h to knock off the absorbed protein. Finally, the concentration of the adsorbed protein was determined with a Micro BCA protein assay kit.

### In Vitro Cell Adhesion Evaluation

Cell adhesion on the surface of the hydrogels was assessed by using L929 murine fibroblasts and RAW246.7 macrophages, which were originally obtained from the American Type Culture Collection (ATCC). L929 cells and RAW246.7 cells were cultured in DMEM medium supplemented with 10% FBS and RPMI 1640 medium supplemented with 10% FBS, respectively, at 37 °C in a 5% CO_2_ atmosphere. First, 1 mL of PLEL solution was added to a 24‐well plate and maintained at 37 °C for 10 min to form a stable hydrogel disk. L929 cells or RAW246.7 cells (2 × 10^4^ cells/well) were seeded on the surface of the PLEL hydrogel disk and incubated for 48 h. The medium was subsequently discarded, and the surfaces of the hydrogel disks were gently rinsed with PBS to remove the unattached cells. The morphology of L929 cells and RAW246.7 cells attached to the surface of the hydrogels was observed under a microscope (Olympus, Tokyo, Japan). In addition, L929 cells and RAW246.7 cells attached to the surface were stained by a live/dead cell staining kit and observed under a fluorescence microscope (Olympus, Tokyo, Japan). The cells seeded on the surface of the tissue culture plate surface (TCPS) were used as a control.

### Antiadhesion Process and Safety of PLEL Hydrogel in Cardiac Adhesion Prevention

The cardiac anti‐adhesion ability of PLEL hydrogel was studied via an established and highly reproducible cardiac injury adhesion model in rats.^[^ ^14^, ^31^ ^]^ Briefly, SD rats were anesthetized in an isoflurane‐containing induction chamber and then mechanically ventilated with 2% isoflurane. After shaving around the area of the incision, the thoracic cavity was accessed via a median sternotomy, and the heart was exposed by tearing the pericardium. The exposed heart surface was subsequently abraded by poking 100 times with a 30 G syringe needle and then exposed to air to dry for 10 min, creating an injured area. PLEL solution (400 µL total) was injected onto the surface of the injured heart. The hydrogel was then allowed to gel fully for 10 min before the chest cavity was closed with 4‐0 polypropylene sutures. At the same time, the rats treated with 400 µL of normal saline were used to observe the process of cardiac adhesion formation.

On days 0, 3, 7, 10, and 14 postsurgery, three rats in each group were subjected to dissection and gross observation. The damaged/repaired cardiac tissue was subsequently obtained. Some of the heart samples were fixed with 4% paraformaldehyde, paraffin‐embedded, sectioned, and subjected to hematoxylin and eosin (H&E) staining and Masson's trichrome staining. The other parts of the obtained heart tissues were fixed immediately with 2.5% glutaraldehyde in PBS. After gradient dehydration with ethanol, the samples were dehydrated in a critical point apparatus, coated with a gold sputter, and then examined by scanning electron microscope (SEM, JSM‐5900LV, JEOL, Japan). In addition, the main organs (heart, liver, spleen, lung, and kidney) from each group were harvested at every timepoint, fixed with 4% paraformaldehyde, paraffin‐embedded, sectioned, and subjected to H&E staining. Images were collected with a microscope slide scanner (PANNORAMIC MIDI, 3DHISTECH) and analyzed with CaseViewer software. Blood samples from each group were also collected and subjected to blood chemistry tests. Samples from the normal rats were used as controls.

At the same time, the cardiac function of the rats in all groups was also assessed on days 3, 7, and 14 after surgery by a Vevo 3100 ultrasound imaging system (Visual Sonics, Canada). M‐mode guided by transthoracic 2D methods was used, and the ejection fraction (EF), fraction shortening (FS), left ventricle internal dimensions at end‐systole (LVIDs) and left ventricle internal dimensions at diastole (LVIDd) were measured. All the measurements were averages of three consecutive cardiac cycles.

### In Vivo Antiadhesion Evaluation in a Rat Cardiac Injury Adhesion Model

To evaluate the effectiveness of the PLEL hydrogel for preventing cardiac adhesion, the above operation procedures for the cardiac adhesion model were repeated in another 50 rats. Animals were randomized into 5 groups, including Model, Injected PLEL hydrogel, Sprayed PLEL hydrogel, HA hydrogel, and Interceed film groups, with ten rats in each group. The injured hearts and adjacent tissues of the rats in the Model group were washed with 400 µL of normal saline, while those of the rats in the Injected PLEL hydrogel, Sprayed PLEL hydrogel, and HA hydrogel groups were treated with 400 µL of PLEL hydrogel (injected or sprayed) or HA hydrogel (injected). The rats in the Interceed film group were treated with an Interceed film (1 × 1 cm^2^) on the surface of the injured heart. After that, the chest cavity was closed with 4‐0 polypropylene sutures. Fourteen days after the surgery, the rats were euthanized and dissected to examine the adhesion level. A standard adhesion scoring system was used to assess the degree of adhesion in a double‐blind manner, as shown in Table 1.^[^ ^4^, ^7^, ^14^ ^]^ Heart, thoracic wall, or cardiac adhesion sites were harvested and fixed with 4% paraformaldehyde subjected to H&E staining, Masson staining, and immunohistochemistry analysis. Blood samples from each group were also collected, and the serum was obtained via centrifugation for the subsequent enzyme‐linked immunosorbent assay (ELISA). In addition, the body weights of the rats were recorded before and 14 days after the surgery.

### In Vivo Antiadhesion Evaluation in a Rat Repeated‐Injury Cardiac Adhesion Model

The PLEL hydrogel was further used in a recurrent cardiac adhesion model after adhesiolysis. The repeated‐injury cardiac adhesion model was established first by creating a primary cardiac abrasion without any treatment. Fourteen days after the first surgery, the rats were anesthetized, and a second thoracotomy was performed. Then, the primary adhesion between the heart and the adjacent tissues was subjected to adhesiolysis via appropriate dissection, followed by abrading the surface of the separated heart again to produce secondary injuries. Animals were randomly distributed into three groups (*n* = 12) to receive the PLEL hydrogel (injected), HA hydrogel, or no treatment, as described previously. Fourteen days after the adhesiolysis, the rats were subjected to adhesion scoring, tissue harvesting, serum collection, and body weight recording as described previously.

### Enzyme‐Linked Immunosorbent Assay (ELISA)

Serum samples were obtained from the rats on the 14th day after the first surgery and adhesiolysis, and then was subjected to the ELISA analysis. Tissue‐type plasminogen activator (t‐PA) and plasminogen activator inhibitor‐1 (PAI‐1) in the serum of the rats were measured with ELISA kits (Abcam, UK) following the instructions.

### Immunofluorescence Analysis

Immunofluorescence (IF) staining was performed to observe the pathological changes in the heart, thoracic wall, and cardiac adhesion after different treatments in the cardiac adhesion model. The fixed adhesion‐related tissues obtained from the rats on the 14th day after the first surgery and adhesiolysis were paraffin‐embedded, sectioned (5 µm), deparaffinized, rehydrated, blocked with goat serum and incubated with the primary antibodies. Specifically, the primary antibodies used in this study included rabbit anti‐rat Serpin E1/PAI‐1 antibody (Novus Biologicals, NBP1‐19773, 1:100), mouse anti‐rat t‐PA antibody (Abcam, H27B6‐ab28374, 1:100), rabbit anti‐rat CD68 (Servicebio, GB113109, 1:200), rabbit anti‐rat CD206 (Servicebio, GB113497, 1:400), rabbit anti‐rat TNF‐α (Affinity, AF7014, 1:200), rabbit anti‐rat TGF‐β (Affinity, AF1027, 1:200), and rabbit anti‐rat IL‐6 (Servicebio, GB11117, 1:200). The tissue sections were subsequently incubated with secondary antibodies, including Alexa Fluor 488‐conjugated goat anti‐rabbit IgG (Servicebio, GB25303, 1:400), CY3‐conjugated goat anti‐rabbit IgG (Servicebio, GB21303, 1:300), CY3‐conjugated goat anti‐rabbit IgG (Bioss, bs‐0296G‐Cy3, 1:200), and FITC‐conjugated goat anti‐rabbit IgG (Bioss, bs‐0295G‐FITC, 1:200). Finally, the slides were counter‐stained with DAPI for 10 min. Images were acquired with a fluorescence microscope slide scanner (PANNORAMIC MIDI, 3DHISTECH) and analyzed with CaseViewer software. Fibrinolytic activity and inflammatory effects were semiquantitatively evaluated by comparing the ratios of the relevant positive areas among the different groups, which were measured via ImageJ software in a double‐blinded manner.

### Statistical Analysis

All the data are expressed as the mean ± standard deviation (SD). Statistical comparisons among the groups were performed via Student's t‐test or ordinary one‐way ANOVA analysis using GraphPad Prism software. A *p*‐value < 0.05 was considered statistically significant for all the statistical analyses.

## Conflict of Interest

The authors declare no conflict of interest.

## Supporting information



Supporting Information

## Data Availability

The data that support the findings of this study are available from the corresponding author upon reasonable request.
