# Supplementary material for: Injectable and Sprayable Thermoresponsive Hydrogel with Fouling‐Resistance as an Effective Barrier to Prevent Postoperative Cardiac Adhesions
Source: Adv Sci (Weinh). 2025 Mar 28;12(23):2500731. doi: 10.1002/advs.202500731 (PMC12199412; doi:10.1002/advs.202500731)
Supplement: Supplementary file 1 — Supporting Information [file ADVS-12-2500731-s001.docx]

Supporting information

**Injectable and Sprayable** **[Thermoresponsive Hydrogel with Fouling-Resistance as an Effective Barrier to](https://www.webofscience.com/wos/alldb/full-record/WOS:000414620600007)** **[Prevent](https://www.webofscience.com/wos/alldb/full-record/WOS:000414620600007)** **[Post-operative Cardiac Adhesion](https://www.webofscience.com/wos/alldb/full-record/WOS:000414620600007)s**

Kun Shi^1^, Tao Li^2,3^, Xulin Hu^1,4^, Wen Chen^1^, Yan Yu^1^, Zhongwu Bei^1^, Liping Yuan^1^, Qi Tong^3^, Jiafeng Liu^1^, Qiang Fan^3^, Yongjun Qian^3,^^[[1]](#footnote-0)^*, Zhiyong Qian^1,^^[[2]](#footnote-1)^*

*^1^ Department of Biotherapy, Cancer Center and State Key Laboratory of Biotherapy, West China Hospital, Sichuan University, Chengdu, 610041, China*

*^2^ Department of Pediatric Cardiac Surgery, West China the Second Hospital, Sichuan University, Chengdu, 610041, China*

^3^ *Department of Cardiovascular Surgery, National Clinical Research Center for Geriatrics, West China Hospital, Sichuan University, Chengdu, 610041, Sichuan, PR China*

^4^ *Clinical Medical College and Affiliated Hospital of Chengdu University, Chengdu University, Chengdu, Sichuan 610081, China*

**This PDF file includes:**

**Figure S1.** Structural characterization of PDLLA-PEG-PDLLA copolymers.

**Figure S2.** Characterization of PLEL micelle (1 wt.%).

**Figure S3.** Cytotoxicity of the PLEL hydrogel.

**Figure S4.** H&E staining of the vital organs (heart, liver, spleen, lung, kidney) in different groups 14 days postoperation.

**Figure S5.** Blood chemistry results of rats in normal group, untreated model group, and PLEL hydrogel-treated group on different days after surgery.

**Figure S6.** The body weight change of rats in different groups 14 days after surgery.

**Figure S7.** Effects of the PLEL hydrogel on the inflammatory response in a rat model of repeated-injury cardiac adhesion model.

**
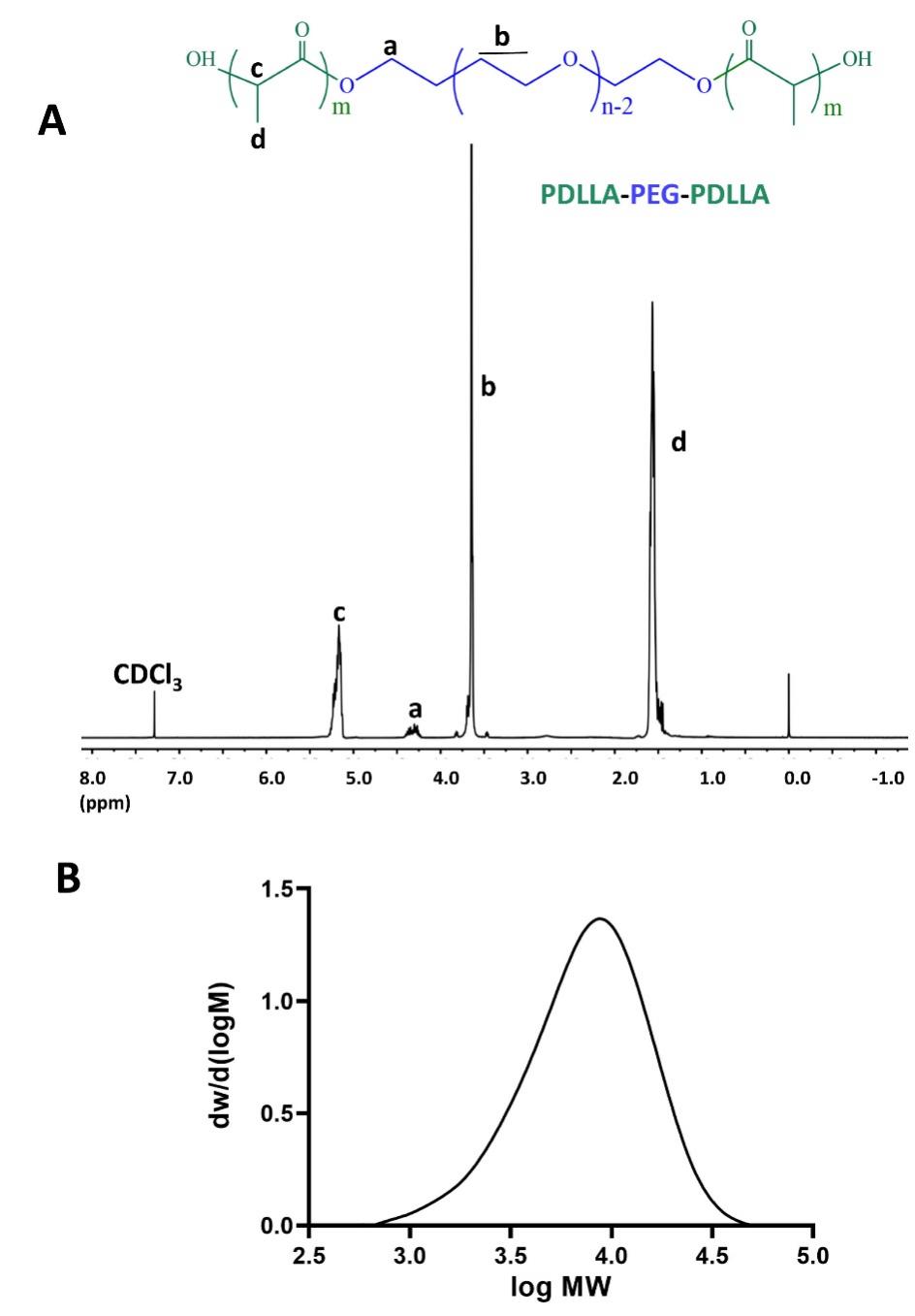
**

**Figure S1.** Structural characterization of PDLLA-PEG-PDLLA copolymers. (A) 1H NMR spectrum of PDLLA-PEG-PDLLA triblock copolymers (solvent: CDCl3). (B) The molecular weight distribution ofPDLLA-PEG-PDLLA triblock copolymers.


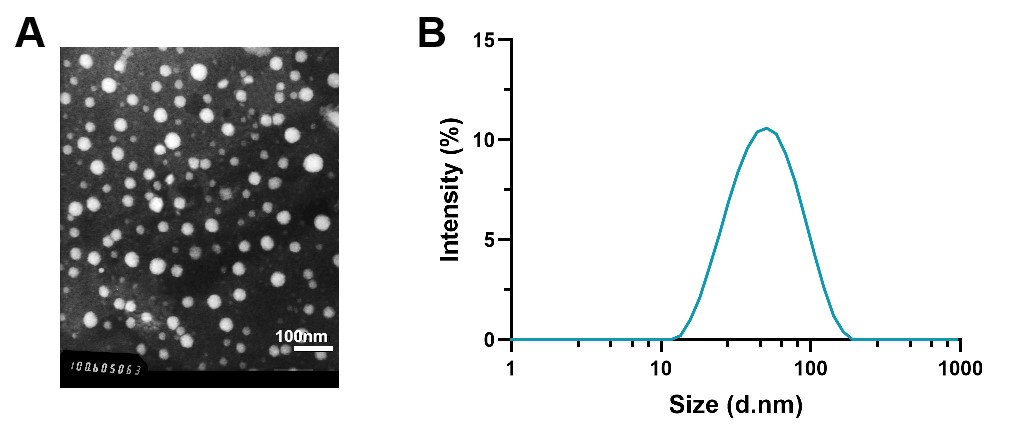


**Figure S2.** Characterization of PLEL micelles (1 wt.%). (A) TEM images of PLEL micelles; (B) The distribution of PLEL micelles measured by DLS.


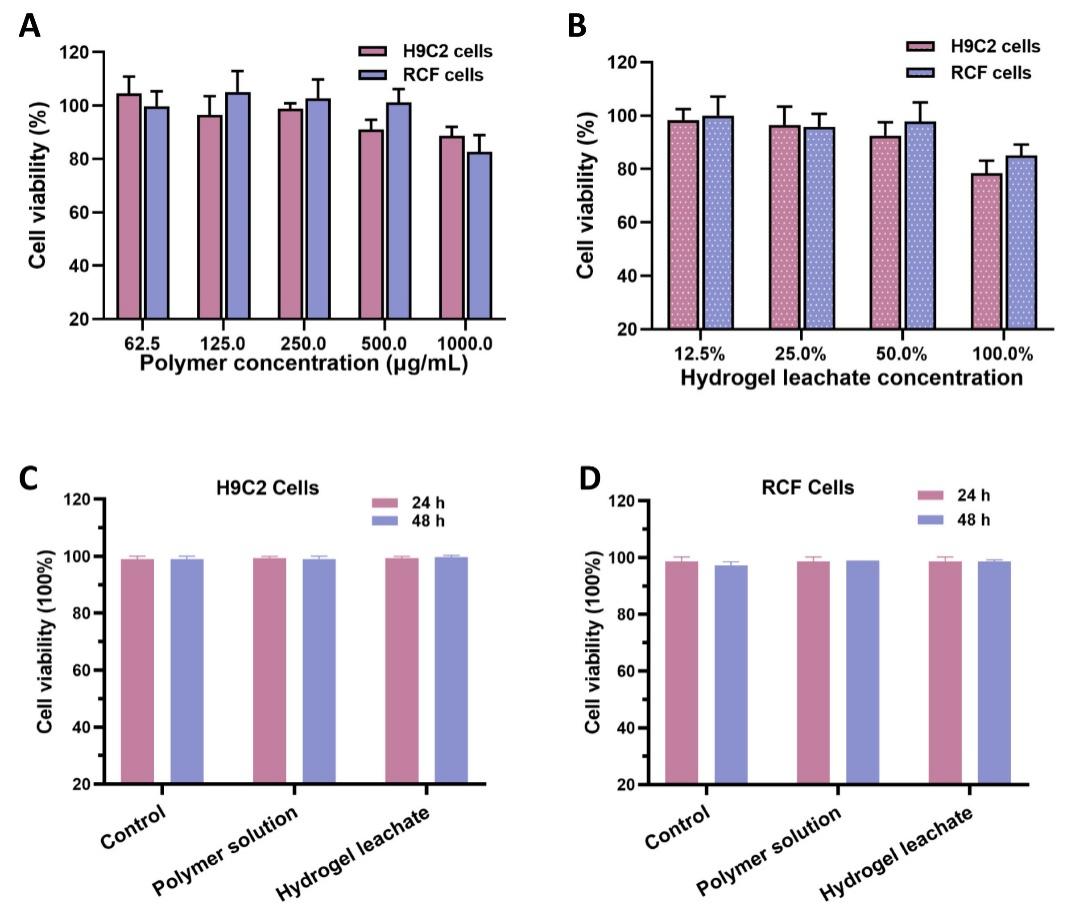


**Figure S3.** Cytotoxicity of the PLEL hydrogel. (A) Cytotoxicity of the PLEL copolymer against H9C2 cells and RCFs after coculture for 48 h. Data are presented as the mean ± SD. (n = 5). (B) Cell viability of H9C2 cells and RCFs after treatment with PLEL hydrogel leachate for 48 h. Data are presented as the mean ± SD. (n = 5). (C) The proportion of living cells analyzed by the Live/dead imaging of H9C2 cells. (D) The proportion of living cells analyzed by the Live/dead imaging of RCF cells.


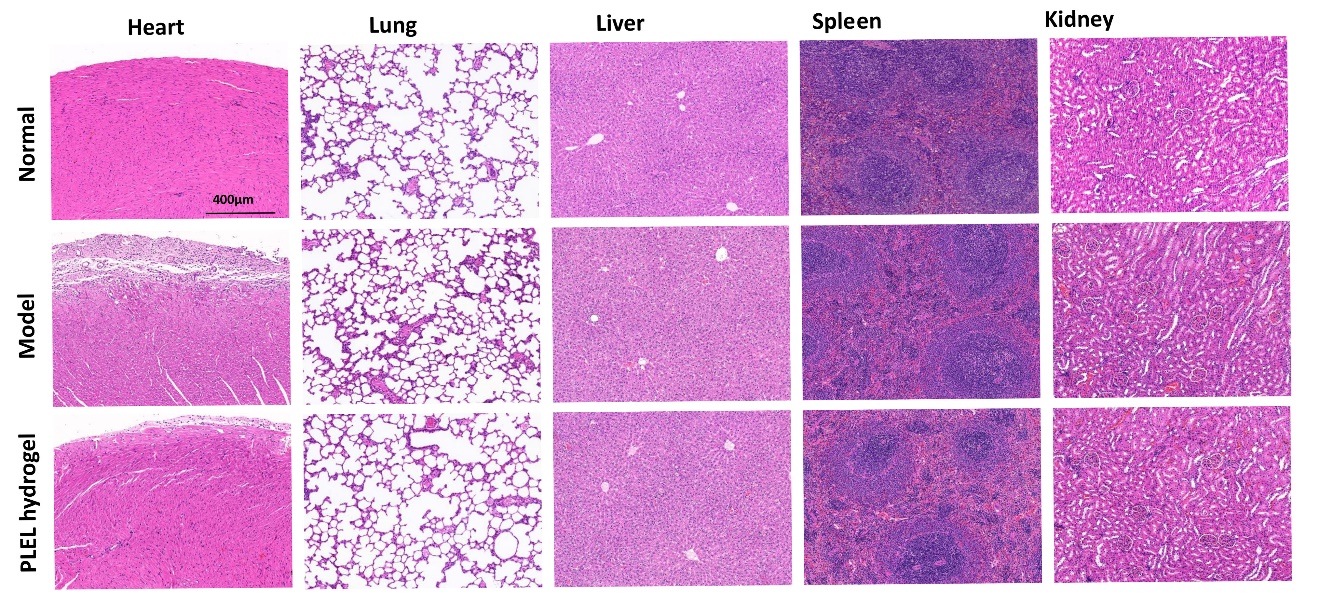


**Figure S4.** H&E staining of the vital organs (heart, liver, spleen, lung, kidney) in different groups 14 days postoperation.


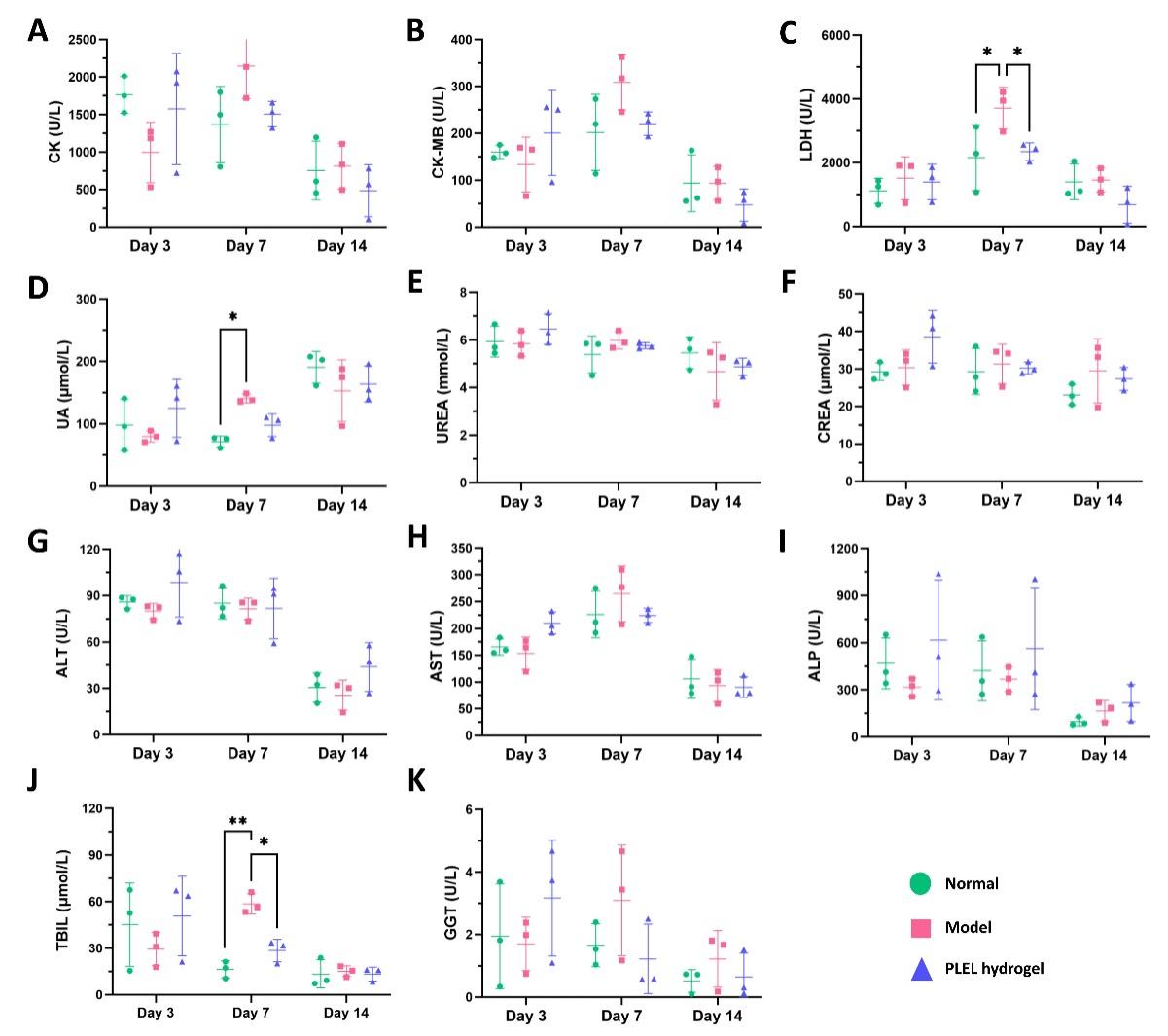


**Figure S5.** Blood chemistry results of rats in normal group, untreated model group, and PLEL hydrogel-treated group on different days after surgery (n = 3). **p* < 0.05, ***P* < 0.01.


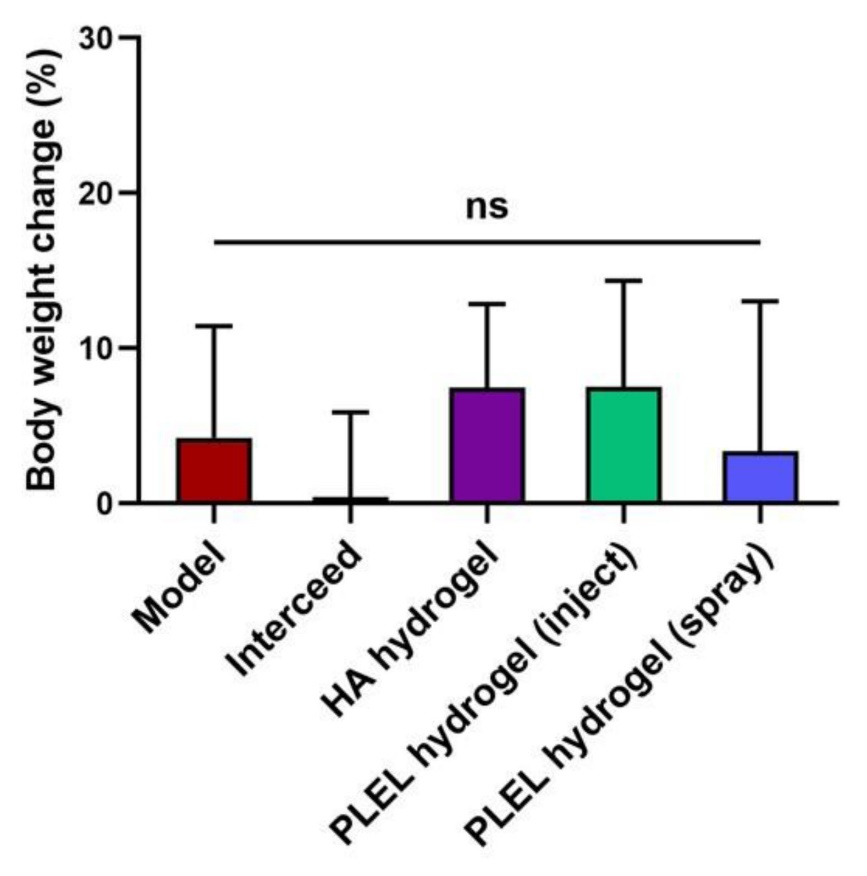


**Figure S6.** The body weight change of rats in different groups 14 days after surgery. The ns means no significant difference.


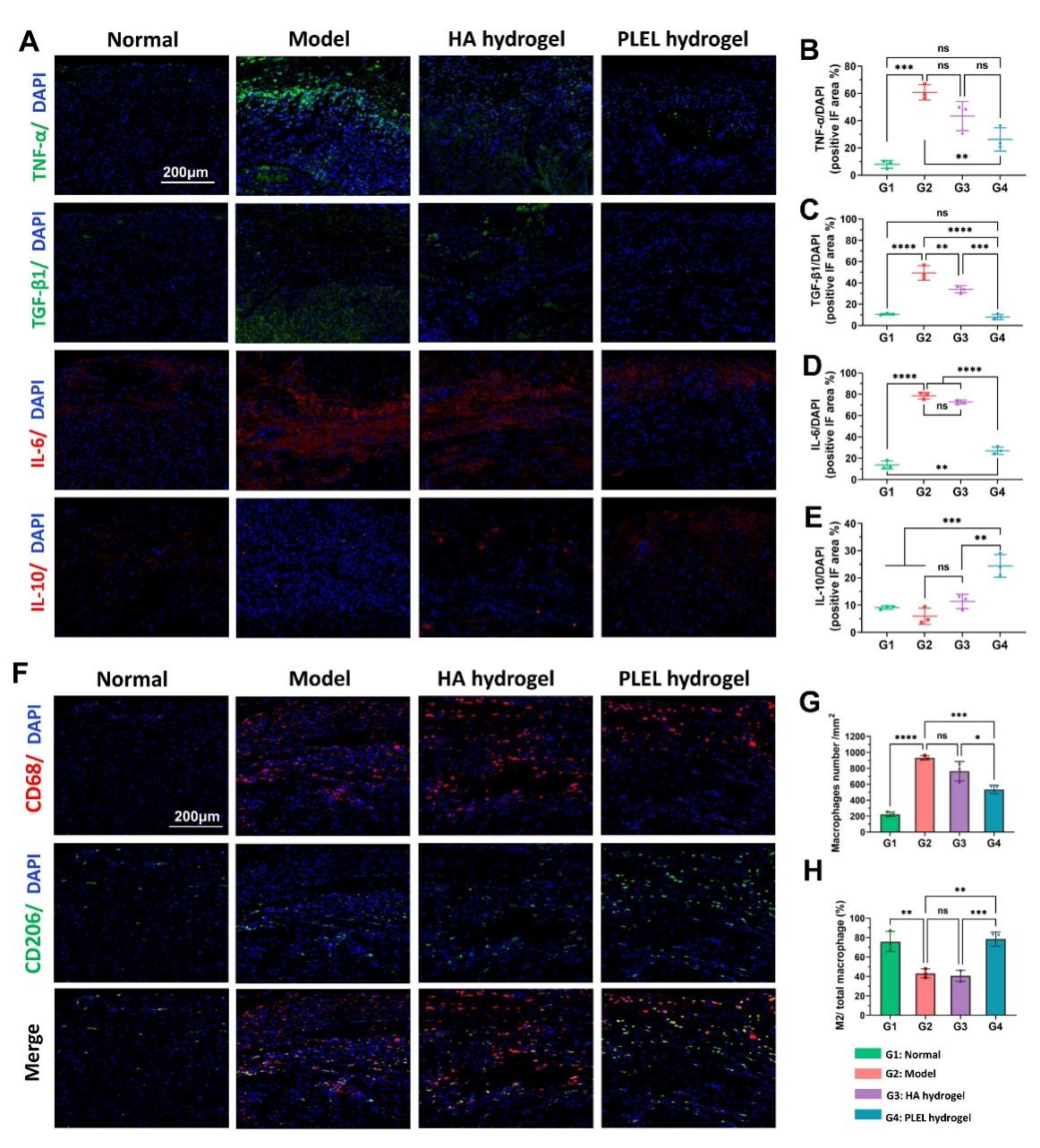


**Figure S7.** Effects of the PLEL hydrogel on the inflammatory response in a rat model of repeated-injury cardiac adhesion model. (A) IF staining images of TNF-α (green), TGF-β1 (green), IL-6 (red), IL-10 (red), and DAPI (blue) in heart and adhesion tissues from different groups two weeks after surgery. (B-E) Fluorescent area ratios of TNF-α/DAPI, TGF-β1/DAPI, IL-6/DAPI, and IL-10/DAPI (n = 3). (F) IF staining images of CD68 (red), CD206 (green), and DAPI (blue) in heart and adhesion tissues from different groups two weeks after surgery. The expression of CD68 represents total macrophages, and CD206 represents M2 macrophages. (G) The number of total macrophages by counting the number of CD68-positive cells (n = 3). (H) The ratio of M2 macrophages labeled with CD206 to total macrophages (n = 3). The ns indicates no significant difference. **p* < 0.05, ***P* < 0.01 and ****P* < 0.001, *****p* < 0.0001.

1. * Corresponding author. [↑](#footnote-ref-0)
2. E-mail addresses: qianyongjun@scu.edu.cn (Y. Qian), zhiyongqian@scu.edu.cn (Z. Qian). [↑](#footnote-ref-1)
